# Broadly neutralizing antibodies for HIV therapy in clinical trials: a systematic review

**DOI:** 10.1186/s40249-026-01471-4

**Published:** 2026-07-02

**Authors:** Jinfang Zhao, Hui Wu, Jiayi He, Yinsong Luo, Jiaye Liu

**Affiliations:** 1 National Clinical Research Center for Hematologic Disease, Peking University People’s Hospital, Peking University Institute of Hematology, No. 11 South Street of Xizhimen, Xicheng District, Beijing, 100044 China; 2 School of Public Health, Shenzhen University Medical School, No. 1066 Xueyuan Avenue, Shenzhen, 518060 China

**Keywords:** HIV-1, Broadly neutralizing antibodies, Pharmacokinetics, Antiviral efficacy, Safety

## Abstract

**Background:**

Lifelong daily antiretroviral therapy (ART) effectively suppresses human immunodeficiency virus type 1 (HIV-1) replication but does not eradicate the virus, underscoring the urgent need for long-acting antivirals and functional cure strategies. Broadly neutralizing antibodies (bNAbs) have emerged as a promising approach for achieving durable HIV-1 remission. In this systematic review, we summarize recent advances in the development of bNAbs for HIV-1 treatment.

**Methods:**

We searched PubMed, Embase, and Web of Science for clinical trials published up to March 22, 2026. We included data evaluating intravenously administered bNAbs, with or without concomitant conventional ART, and comparing them with placebo, ART or no intervention. These data were used to evaluate the pharmacokinetics, antiviral efficacy, resistance profiles, immunologic effects, and safety of intravenously administered bNAbs.

**Results:**

LS-modified bNAbs extended half-life by 2- to 5-fold relative to their parental counterparts (e.g., VRC01LS: 71 vs 15 days), although viremia reduced half-life by 20–40%. In viremic participants harboring bNAb-sensitive virus, monotherapy achieved viral load (VL) reductions of 0.93–1.8 log₁₀ copies/ml, with rebound occurring within approximately 4–8 weeks, whereas combinations regimens achieved declines of up to 2.04 log₁₀ copies/ml and delayed rebound to 15–33 weeks. No consistent reduction in total reservoir size was observed, although early intervention and baseline viral sensitivity appeared to limit reservoir expansion. Resistance emerged through epitope-proximal mutations, with cross-resistance observed mainly among antibodies targeting shared epitope classes. Overall, bNAbs were well tolerated, with rare discontinuations (0.4%) and low immunogenicity.

**Conclusions:**

LS-engineered bNAbs exhibit a longer half-life than their parental antibodies. Combination regimens achieve greater VL reductions and a longer delayed rebound compared with monotherapy, indicating that LS-modified multi-epitope bNAb cocktails are promising for long-acting HIV-1 remission. To become clinically competitive, larger resistance-guided trials are needed to extend dosing intervals, improve resistance mitigation, and define optimal integration with other long-acting therapies.

**Graphical Abstract:**

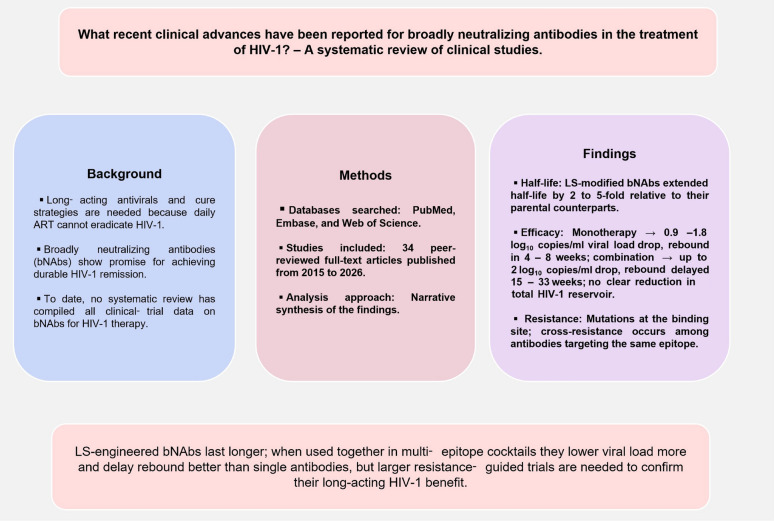

**Supplementary Information:**

The online version contains supplementary material available at 10.1186/s40249-026-01471-4.

## Background

Since its identification in 1983 as the causative agent of acquired immunodeficiency syndrome (AIDS), HIV-1 continues to pose a major global health challenge, and no clinically implementable strategy is currently available to achieve complete eradication or a functional cure [1, 2]. Antiretroviral therapy (ART) remains the cornerstone of HIV-1 management [3]. With the number of people living with HIV-1 (PLWH) on ART worldwide reaching 31.6 million in 2024, it has transformed AIDS into a chronic, manageable condition [3]. However, ART does not eliminate the virus from the latent reservoir [1], and treatment interruption usually leads to rapid viral rebound, necessitating lifelong therapy that is often complicated by drug resistance, toxicity, and adherence challenges [4, 5].

The major barrier to a cure is the persistence of the latent viral reservoir, which is composed predominantly of resting CD4^+^ T cells harboring replication-competent proviral deoxyribonucleic acid (DNA). These reservoir cells, alongside long-lived immune cells such as macrophages and microglia, are not eradicated by ART and continue to sustain the potential for viral reactivation [1]. Earlier attempts using first-generation monoclonal antibodies (e.g., 2G12, 2F5, and 4E10) showed limited efficacy and were frequently associated with rapid viral escape [6, 7]. Over the past decade, the development of second-generation broadly neutralizing antibodies (bNAbs) has revitalized interest in immunotherapeutic strategies for HIV-1 remission and cure. These bNAbs target conserved regions of the HIV-1 envelope (Env) trimer, including the CD4 binding site (CD4bs, e.g., VRC01, 3BNC117, VRC01LS, VRC07-523LS, VRC07-523, and N6LS), the V1/V2 apex (e.g., PGDM1400 and PGDM1400-LS), the V3 glycan supersite (e.g., PTG121, PTG121.414-LS, and 10–1074), the fusion peptide, the membrane-proximal external region (e.g., 10E8-LS), and other relatively inaccessible epitopes such as the silent face and subunit interface [8, 9]. Additionally, bispecific and trispecific antibodies have been developed to further enhance neutralization breadth and potency [8].

Beyond direct neutralization, bNAbs can exert antiviral activity through Fc-dependent effector functions, including antibody-dependent cellular cytotoxicity (ADCC), and may also enhance HIV-specific T-cell responses through engagement of Fcγ receptors [8, 10]. Together, these mechanisms not only enable viral suppression but also offer the potential to modulate the viral reservoir, particularly when bNAbs are administered during structured treatment interruption or after early ART initiation.

Despite these promising attributes, no bNAb regimen has yet achieved a cure. To date, no comprehensive systematic review has synthesized the findings from clinical trials evaluating intravenously administered bNAbs in both HIV-negative individuals and PLWH. To address this gap and inform future therapeutic strategies, we conducted a systematic review of clinical trials of intravenous bNAb therapy, with the aim of assessing their pharmacokinetics, antiviral efficacy, resistance patterns, immunologic effects, and safety profiles.

## Methods

### Search strategy and selection process

This systematic review was conducted in accordance with the methodological guidance outlined in the Cochrane Handbook for Systematic Reviews of Interventions and reported according to the Preferred Reporting Items for Systematic Reviews and Meta-Analyses (PRISMA) 2020 statement. The study protocol was prospectively registered with PROSPERO (CRD420250653908). We conducted a systematic search of PubMed, Web of Science, and Embase for English-language studies published up to March 22, 2026, using the following terms: (“HIV” OR “human immunodeficiency virus” OR “AIDS” OR “acquired immunodeficiency syndrome”) AND (“antibod*” OR “monoclonal antibody” OR “neutralizing antibody” OR “broadly neutralizing antibody” OR “bNAb*”). To minimize language and regional publication bias, we additionally searched major Chinese databases, including CNKI, Wanfang Data, and SinoMed. No additional eligible clinical trials were identified. Detailed search strategies are provided in the appendix (Additional file 1: Table S1). No geographic restrictions were applied.

### Inclusion and exclusion criteria

Eligible studies included clinical trials evaluating intravenously administered second-generation bNAbs, with or without concomitant conventional ART, and comparing them with placebo, ART or no intervention. Studies were excluded if they did not align with the therapeutic focus of this review, such as those primarily investigating HIV-1 prevention or epidemiological prevalence. We also excluded non-original studies and non-human research, including meeting abstracts, letters, reviews, commentaries, opinion pieces, case reports, meta-analyses, animal models, and in vitro experiments. Because this review focused on the therapeutic use of bNAbs for HIV-1, and to maintain a homogeneous dataset while minimizing variability in exposure-response relationships, we restricted the analysis to studies of intravenously administered bNAbs. Studies involving intramuscular or subcutaneous administration, vector-based antibody delivery, co-infections, or combination interventions outside the scope of this review were excluded.

### Identification of studies and data extraction

The selection process was carried out in several steps. Two reviewers (JFZ and HW) independently screened titles, abstracts, and full texts according to the predefined inclusion and exclusion criteria. Discrepancies were resolved through discussion or consultation with a third reviewer (JYL). For each article that was excluded, we documented the specific reason for its exclusion. Finally, all search results were combined, duplicate records were identified, verified, and removed.

A standardized data extraction form was developed a priori to collect study characteristics and participant-level variables. Extracted data included first author, year of publication, sample size, participant category (including HIV-1-negative individuals, PLWH off ART, PLWH on ART, and ART-naïve PLWH), sex, country, ethnicity, body weight, baseline viral load (VL) (day 0), baseline CD4^+^ T-cell count (day 0), duration of ART, length of follow-up, and study design. Additional outcomes extracted included pharmacokinetic parameters, IgG gamma marker (GM), anti-drug antibody (ADA), neutralizing activity, therapeutic effectiveness, resistance profiles to bNAbs, and immunological responses following bNAbs administration. Articles meeting the inclusion criteria were retained in the synthesis even if some variables (e.g., body weight, resistance data, or ART duration) were missing.

### Study quality assessment and the risk of bias

The risk of bias and overall methodological quality of each study were evaluated according to its design. Randomized controlled trials (including the randomized arms of partially randomized trials) were assessed with the Cochrane Risk of Bias 2 (RoB 2) tool, focusing on virologic efficacy and safety outcomes. Non‑randomized comparative studies were appraised using the Risk of Bias in Non‑randomized Studies of Interventions (ROBINS-I) tool, and single-arm, open-label early-phase trials without a comparator were evaluated with the NIH Quality Assessment Tool for Case Series Studies, which grades studies as good, fair, or poor. For RoB 2, studies were classified as low risk, some concerns, or high risk of bias; for ROBINS‑I, they were judged as low, moderate, serious, or critical risk of bias. Two reviewers (JYH and HW) performed the assessments independently; any disagreements were resolved through discussion or, when necessary, by consulting a third reviewer (JFZ). Detailed ratings are presented in Additional file 2: Table S2.

### Certainty assessment

The certainty of evidence for each outcome domain was evaluated using the Grading of Recommendations Assessment, Development and Evaluation (GRADE) framework. Because the included studies were early‑phase trials that were clinically and methodologically heterogeneous, a quantitative meta‑analysis was inappropriate; therefore, GRADE was applied as a structured, narrative assessment rather than a pooled‑effect analysis. The domains assessed were safety, pharmacokinetics, antiviral efficacy, reservoir dynamics, and immunologic responses. For each domain, certainty was graded as high, moderate, low, or very low, taking into account risk of bias, inconsistency, indirectness, imprecision, and potential reporting bias. Two reviewers (JFZ and JYH) performed the assessments independently, and any disagreements were resolved through discussion or, when necessary, by consulting a third reviewer (YSL). Detailed ratings are provided in Additional file 3: Table S3.

### Data synthesis strategy

Due to significant heterogeneity among the studies in terms of study designs, study populations, and research protocols, a comprehensive narrative synthesis was performed to address the review’s objective. The study findings are presented in tabular and figure form, highlighting the pharmacokinetics, antiviral efficacy, resistance profiles, immunologic effects, and safety of intravenously administered bNAbs.

### Treatment strategies

Three treatment strategies were identified across the included trials: monotherapy, dual-combination, and triple-combination therapy. These strategies involved up to ten different bNAbs and/or placebo, administered as single or multiple intravenous infusions at doses ranging from 1 to 40 mg/kg under dose-escalation protocols. Monotherapy was defined as administration of a single bNAb, either alone or in combination with placebo or ART. Dual and triple-combination strategies were defined as the simultaneous administration of two or three bNAbs, respectively. In trials involving analytic treatment interruption (ATI), participants were followed after their first or last infusion of bNAbs or placebo. One study evaluated early intervention with 3BNC117 in newly diagnosed individuals who had recently initiated ART. Another trial implemented a three-phase design consisting of: (1) a lead-in phase with concurrent ART and bNAb administration, (2) a bNAb-only phase following ART interruption, and (3) an ART re-initiation phase, in which participants restarted ART either at week 24 or upon reaching a predefined VL threshold (≥ 400 copies/ml).

### Baseline viral sensitivity to administered bNAbs

Baseline viral sensitivity to the administered bNAbs was assessed using either phenotypic neutralization assays or, when phenotypic testing was not feasible, validated genotypic resistance-prediction algorithms. In the phenotypic approach, autologous Env-derived pseudoviruses were tested against the relevant bNAb(s), and sensitivity was defined according to the inhibitory concentration thresholds reported in the original studies [e.g., inhibitory concentration 50% (IC50), IC80, or IC90]. When phenotypic results were unavailable, genotypic prediction algorithms based on resistance-associated Env mutations were used to classify viruses as sensitive or resistant according to the original study definitions. Because sensitivity thresholds and reporting metrics varied across studies, we retained the study-specific definitions where available and synthesized them within a common framework of baseline susceptibility versus resistance.

### Definitions of therapeutic effectiveness and effect measures

Treatment outcomes were categorized into three commonly used virologic endpoints, as reported in the included trials. Although exact definitions varied slightly across studies, the following criteria were applied for synthesis: complete viral suppression was generally defined as plasma HIV-1 RNA below the lower limit of quantification of standard assays, most commonly reported as < 20 or < 50 copies/ml; viral rebound typically referred to a confirmed increase in plasma HIV-1 RNA to > 200 copies/ml following a period of suppression to undetectable levels; and loss of virologic control was commonly defined as a confirmed plasma HIV-1 RNA level ≥ 1000 copies/ml after prior suppression. Where available, we adopted the original definitions used in each study; however, when such definitions were not explicitly stated, the above criteria were used as reference standards for cross-study comparison.

Because of the heterogeneity in these trials, effect measures were summarized as reported in the original studies. For pharmacokinetic outcomes, we extracted half-life, peak concentration, trough concentration, and antibody concentrations at prespecified time points. For virologic outcomes, we extracted log10 changes in plasma HIV-1 RNA, proportions of participants achieving complete viral suppression, time to viral rebound, and time to loss of virologic control. For safety outcomes, we extracted the number of adverse events, and bNAbs-related AEs. For resistance, reservoir, and immunologic outcomes, we summarized the measures, assays, and definitions used in the original studies.

### Patient and public involvement

No patients were involved in the conceptualisation or conduct of this study due to the nature of the study as a systematic review.

## Results

### Study selection and screening process

**Fig. 1:**
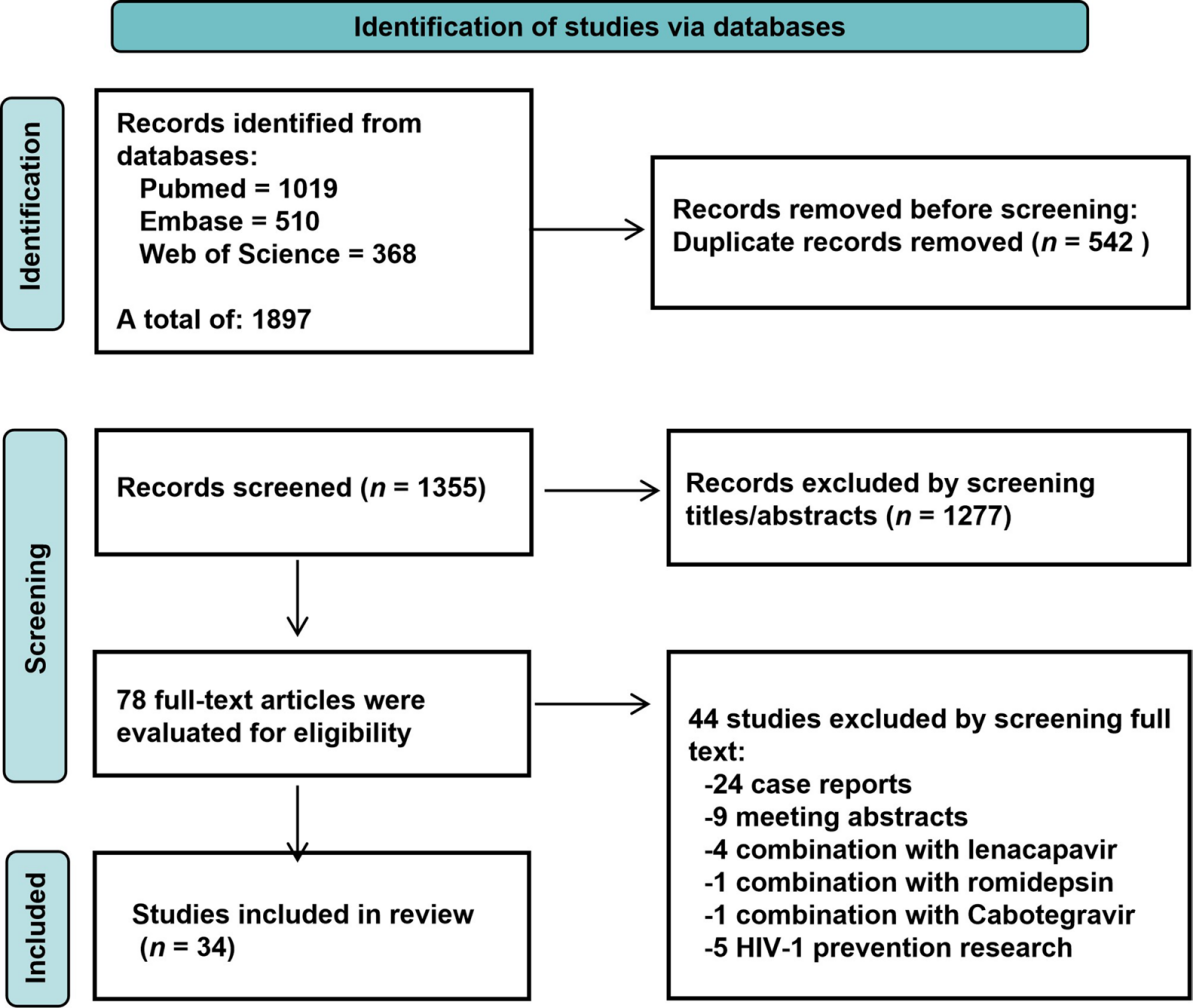
Flow chart of the database search and study selection process

A total of 1897 records were initially identified through database searches (Fig. 1). After duplicate removal, 542 unique records remained. Screening of titles and abstracts resulted in the exclusion of 1277 records deemed irrelevant. The full texts of 78 articles were then assessed for eligibility, of which 44 were excluded for not meeting the inclusion criteria. Ultimately, 34 full-text articles were included in the systematic review (Fig. 1).

### Characteristics of included bNAb clinical trials

Data were extracted from 34 peer-reviewed full-text articles published between 2015 and 2026 [11–44]. Of these, 9 were conducted exclusively in HIV-negative individuals and 25 involved PLWH, including 11 studies in aviremic individuals receiving suppressive ART, 4 in viremic individuals, and 6 in mixed-population cohorts (Table 1 and Additional files 4–5: Tables S4–S5). Four studies were extensions of four earlier studies. Overall, the included studies comprised 13 randomized controlled trials (RCTs), 3 partial RCTs, 23 open-label trials, 3 blinded trials, and 4 partial open-label trials (Tables 1, 2, 3).

**Table 1:** Overview of included studies in HIV-negative individuals

First author (year)	Country	*n*	Male (%)	Age (years)	Body weight (kg)	Study design/trial phase	Follow-up (weeks)	Hispanic (%)	BNAb target	Schedule	bNAb-related AEs and SAEs	IgG GM	ADA	Neutralizing activity
Ledgerwood, J E [11] (2015)	USA	18	72	(21, 50)#	76	Open-label/Phase 1a	144	0	The CD4-binding site of the HIV envelope	VRC01: 5 mg/kg × 2 doses; 20 mg/kg × 2 doses; 40 mg/kg × 2 doses	One case of elevated ALT (54 IU/L) in the 5 mg/kg group (mild)	No	No	VRC01 retained its neutralizing activity in serum up to day 56
Mayer KH [12] (2017)	USA	64	50	(18, 50)#	NA	RCT, open-label/Phase 1a	32	13	The CD4-binding site of the HIV envelope	VRC01: 40 mg/kg then 20 mg/kg q4w × 5 doses; 40 mg/kg q8w × 3 doses; 10 mg/kg q8w × 3 doses; 30 mg/kg q8w × 3 doses	NA	NA	No	VRC01 maintained serum neutralizing activity at 24 weeks
Gaudinski MR [13] (2018)	USA	19	68	(21,50)#	73	Open-label/Phase 1a	24	11	A modified VRC01 by enhancing binding to the neonatal Fc receptor	VRC01LS 5 mg/kg × 1 dose; 20 mg/kg × 1 dose; 40 mg/kg × 1 dose; 20 mg q12w × 3 doses	One case of diarrhea in the 5 mg/kg group (mild)	No	No	VRC01 maintained serum neutralizing activity through 24 weeks
Gaudinski MR [14] (2019)	USA	18	44	30* (21, 50)	73	Open-label/Phase 1a	24	11	A clonal variant of VRC01	VRC07–523LS 1 mg/kg × 1 dose; 5 mg/kg × 1 dose; 20 mg/kg × 1 dose; 40 mg/kg × 1 dose; 20 mg/kg q12w × 3 doses	One case of grade 1 dizziness in 40 mg/kg group; One each of grade 1 abdominal pain and grade 2 infusion reactions in 20 mg/kg group	No	No	VRC07‑523LS showed stronger neutralization and broader coverage than VRC01‑LS, even at lower serum levels
Sobieszczyk ME [15] (2023)	USA	27	41	26 (19,50)	68(51, 86)	Partial RCT, open-label/Phase 1a	48 and 64	11	PGT121: the HIV-1 Env protein: V3 glycan; PGDM1400: the V2 glycan	20 mg/kg × 1 dose PGT121 + VRC07–523LS; PGDM1400 + VRC07–523LS; 10–1074 + VRC07–523LS; 20 mg/kg × 2 doses PGT121 + PGDM1400 + VRC07–523LS	One case of mild and episodic bilateral hand paraesthesia in 10–1074 + VRC07-523LS group (20 mg/kg, each); One case of mild infusion-related reaction (chills, mild upper back muscle pain, mild joint pain in wrists, and a mild headache in PGDM1400 + VRC07-523LS group (20 mg/kg, each)	NA	No	BNAbs retained neutralizing activity in serum
Edupuganti S [16] (2025)	USA	20	30	(22,41)#	(57, 98)	RCT,, open-label/Phase 1a		54 (31–60)	PGT121.414.LS: A modified version of PGT121 with changes in the Fc region: methionine replaced by leucine and asparagine replaced by serine	PGT121.414.LS: 3 mg/kg × 1 dose; 10 mg/kg × 1 dose; 30 mg/kg × 1 dose; PGT121.414.LS + VRC07-523LS: 20 mg/kg q16w × 3 doses	PGT121.414.LS + VRC07-523LS (20 mg/kg, each): 6 mild infusion reactions in 3 individuals	NA	No	BNAbs retained neutralizing activity in serum The dual‑combination regimen shows greater magnitude and broader coverage than a single‑agent regimen
Walsh SR [17] (2024)	USA and Switzerland	59	44	(18, 50)#	(19.0, 43)	RCT, open-label/Phase 1a	More than 144	14	An engineered variant of a clonal variant of VRC01	VRC07-523LS q4m × 5 doses: 2.5 mg/kg; 5 mg/kg; 20 mg/kg	NA	NA	No	VRC07-523LS retained neutralizing activity in serum
Wu RL [18] (2025)	USA	14	50	28*(22, 40)	76*(51.4, 92.7)	Open-label/Phase 1a	24	14	CD4-targeted, modified with an LS mutation	N6LS: at 5, 20 and 40 mg/kg × 1 dose; 20 mg/kg q12w × 3 doses	Single dose 20 mg/kg: one case of neutropenia (moderate); Repeat doses 20 mg/kg: One case of diarrhoea (severe); one case of mild ALT elevation	NA	No	N6LS retained neutralizing activity in serum
Seaton KE [19] (2025)	USA	9	33	(24, 38)#	(57.7, 95.1)	RCT, partial open-label/Phase 1a	24	0	HIV envelope V2 apex with a lysine-serine modification	PGDM1400LS at 5, 20 and 40 mg/kg × 1 dose	Two cases of mild malaise or fatigue, one each of mild headache and moderate headache in 20 mg/kg group, one case of mild nausea in 5 mg/kg; one case of mild headache in 40 mg/kg group	NA	No	PGDM1400LS retained neutralizing activity in serum

The “mean” is denoted by *, and the range is denoted by ^#^

*q8w* once every eight weeks, *q12w* once every twelve weeks, *RCT* randomized controlled trial, *NA* not available, *AEs* adverse effects, *SAEs* serious sdverse event, *ADA* anti-drug antibody, *IgG GM* IgG gamma marker, *bNAb* broadly neutralizing antibody

**Table 2:** Overview of studies in PLWH treated with individual bNAbs

First author (year)	Country	Study design	Schedule	bNAb-related AEs and SAEs	Effectiveness (log _10_ copies/ml)	Resistance	Immune response
Caskey M [20] (2015)	USA Germany	Open- label/Phase 1b	3BNC117: at 1, 3, 10 or 30 mg/kg × 1 dose	Mild to moderate, such as rhinorrhea and/or cough, malaise, headache, diarrhea, myalgia/arthralgia, and sore throat	VL reduction: 1–3 mg/kg: small effect 10 mg/kg: a mean reduction of 1.31 log (1.25 to 1.36 log_10_) were observed in 2/3 participants 30 mg/kg: a mean reductions of 1.48 log (0.8 to 2.5 log_10_) were observed in all 8 participants (nadir at a median of 7 days, significant from days 4 to 28)	3BNC117 leads to selection for high-level resistance in some but not all cases 3BNC117 selected the following mutations: G459D, Q363H (2E1), S461D (2E2), S274Y (2E2), and a lengthened V5 loop	No alteration in T cell counts
Schoofs T [21] (2016)	USA Germany	NA	Control viremic PLWH 3BNC117 at 1, 3, 10, or 30 mg/kg × 1 dose	NA	3BNC117 improved heterologous tier 2 virus neutralization in most participants, but less so in ART-treated individuals	At week 4, most individuals showed increased resistance to 3BNC117, indicating viral escape Significant shifts in sequence diversity were observed in viremic subjects	Viremic individuals treated with 3BNC117 generated antibodies against both sensitive and resistant viruses
Stephenson KE [22] (2021)	USA	RCT, open- label/Phase 1b	PGT121 or placebo: at 3, 10, or 30 mg/kg × 1 dose in HD and PLWH with ART; at 30 mg/kg in individuals not on ART	In HIV-1-negative subjects: one case of grade 2 fatigue, one case of grade 2 gastroenteritis, and one case of grade 1 headache; In viremic PLWH: one case of grade 1 fatigue	1. High VL reduction: Sensitive patients (4/7): a median reduction of 1.77 log_10_; nadir at a median of 8.5 days; returned to baseline by day 28 2. VL reduction: All 4 sensitive patients reached < 40 copies/ml by day 7; 2 rebounded with PGT121-resistant virus by day 28, while the other 2 stayed suppressed until rebound at ≥ 168 days	1. Rebound viruses: fully or partially sensitive to PGT121 2. Resistance mutations observed: Glycan Loss: N332 (single variant) and N332/N334 (multiple variants) V3 Loop (GDIR Motif): D325N, D325K Glycan Addition: N413 Combined: D325 mutations with N332/N334 glycan loss; D325 mutations combined with N332 sequon changes (NIS to DIS or NIN) 3. Rebound viruses: resistant to V3-specific antibody 10–1074; sensitive to non-V3 antibodies	No alteration in CD4^+^ T cell counts No T-cell immunity improvement
Caskey M [23] (2017)	USA	Open- label/Phase 1b	10–1074: at 3, 10 and 30 mg/kg × 1 dose	Five cases of headache at 10/30 mg/kg; One case of dizziness at 3 mg/kg; One each of abdominal pain and malaise/fatigue at 10 mg/kg; One each of elevated total bilirubin and pruritus at 30 mg/kg 2. All these AEs were mild	VL reduction: 10 mg/kg (3/3): a mean reduction of 1.4 log10 (1.08–1.56 log_10_; nadir at 7–9 days; viral RNA returns to baseline in 3–4 weeks 30 mg/kg (11/13 sensitive strains): a mean reduction of 1.52 log_10_; nadir at an average of 10.3 days; significant reduction from day 3 to 27	Resistance quickly developed due to mutations at position 332 (N332 or S334) or changes at D/N325 in the 324G (D/N)IR327 motif Resistance to 10–1074 did not relate to resistance to antibodies targeting other Env sites Resistance to 10–1074 was associated with PGT121	T cell subsets were unchanged
Lynch RM [24] (2015)	USA	Open- label/Phase 1b	VRC01: PLWH on ART: 1–2 doses at 1, 5, 20, and 40 mg/kg; Naive: 40 mg/kg	No	VRC01 does not reduce cell-associated HIV in individuals on effective ART VL reduction: 1.1–1.8 log_10_ in 6/8 patients, return to baseline ~ 10 days; 2/8 resistant at baseline. VLs significantly decreased between days 3–21, with a nadir at day 9 2 low VLs (745 and 237 copies/ml) maintaining < 20 copies/ml for over 20 days, then returning to baseline by days 42–56 No reduction in HIV DNA levels in blood CD4 T cells	Selection for certain residues within the VRC01 epitope: In or near the β20/β21 portion of the VRC01 epitope Within loop D and loop V5: at positions 280, 429 and 462 Multiple residue changes in V5 loop	NA
Happe M [25] (2025)	USA	RCT, open- label/Phase 1b	40 mg/kg × 1 dose VRC01LS or VRC07-523LS without initiate ART for a minimum of 14 days	VRC07-523LS: One mild transient paresthesia and one severe transient decrease in neutrophil count (neutrophil count = 0.683 × 10⁹ L⁻^1^) VRC01LS: No	VL reduction: VRC01LS: a mean reduction of 0.8 log_10_ (1.8 log_10_ in individuals with sensitive viruses) VRC07-523LS: a mean reduction of 1.7 log_10_	Under monotherapy, when the virus is not fully suppressed, resistance to the infused antibody develops	CD4^+^ T cell counts increased after infusion, with a median rise of 59 cells/μL for VRC01LS (average 12 days) and 82 cells/μL for VRC07-523LS (average 10 days)
Riddler SA [26] (2018)	USA	RCT, double-blind/Phase 1b	VRC01 40 mg/kg at weeks 0 and 3, then placebo at weeks 6 and 9; or the reverse	One grade 2 rash with grade 1 pruritus; one grade 1 rash; and three flu‑like symptoms	No effect on plasma viremia, cellular HIV-1 RNA/DNA levels, or stimulated virus production from CD4^+^ T cells	NA	NA
Scheid JF [27] (2016)	USA	Open- label/Phase IIa	3BNC117 30 mg/kg and ATI 2 d after the first infusion: Group A: day 0 and 21; Group B: day 0, 14, 28 and 42		1. Viral rebound: a mean of 6.7 vs 9.9 weeks in group A vs B, and 8.4 weeks for all participant 2. 6/13 (46%) subjects remained suppressed until at least 9 weeks after ATI 3. 3BNC117 restricted the outgrowth of viral genotypes from the latent reservoir	Resistant variants emerged (e.g., Loop D [274F, 282R], A281D, serine at 456, atypical residues at 282) 3BNC117 rarely causes 10–1074 resistance during rebound	During ATI, mean CD4^+^ T cell counts declined by 127 cells/mm^3^, with increased HIV-1-specific T cell responses observed at 12 weeks
Bar KJ [28] (2016)	USA	Open- label/Phase Ib	VRC01 40 mg/kg: A5340: −1 week before and 2 and 5 weeks after ATI; ATI 1 week after the first infusion NIH: −3 days before ATI, 2 and 4 weeks after ATI, then monthly up to 6 months	A5340: Two grade 1 AEs were reported in 2 participants; an IV site induration and itchiness of the left antecubital fossa	Rebound: A5340: a mean of 4 weeks (12/13) vs. NIH: 6 weeks (10/10) Rebound at 4 weeks: A5340 (38%); NIH (80%)	VRC01 exerted selective pressure on rebounding virus Resistance emerged mainly in or near the VRC01 epitope, especially in the V5 and CD4-binding loops	One participant had a decrease of more than 30% from the baseline CD4 T-cell count
Crowell TA [29] (2019)	Thailand	RCT, double-blind/ Phase II	VRC01 (40 mg/kg) or placebo q3w for 24w + day0 ATI	All infusion-related AEs had mild severity except for one moderate infusion site bruising and one severe generalized urticaria in the VRC01 arm	Complete HIV-1 control: Placebo at 14 days, VRC01 at 29 days Loss of viral control: Placebo at 14 days, VRC01 at 33 days VRC01 group had fewer RNA^+^ CD4^+^ T cells at ART restart, but not significantly	NA	Median CD4 change (ART interruption to resumption): −29 cells/μL VRC01 group had lower Ki67^+^Bcl-2^+^ CD8^+^ CD38^+^ HLA-DR^+^ CD8^+^ T cells and MN-specific ADCP at ART resumption
Cale EM [30] (2020)	Thailand	NA	NA	NA	Participants who showed a faster decay of VRC01 in serum rebounded more rapidly Higher VRC01 sensitivity delayed rebound	The lack of selection for VRC01 resistance during ATI in acute-like viruses	NA
Gunst JD [31] (2022)	Denmark and England	RCT, open-label/Phase 1b/2a	Group:ART; ART + 3BNC117 (30 mg/kg at d7 and 21 after ART initiation), ATI started at d400	A total of 29 AEs: 27 grade 1 and two grade 2; The most common AEs: fatigue (*n* = 10) and headache (*n* = 7)	Faster HIV-1 RNA decay for 3BNC117-sensitive viruses from day 10–24, with a reduction of 16.4% compared to 10% in the ART-only group Enhanced clearance of HIV-1-infected cells, particularly central memory cells Greater reduction in intact proviruses in pre-ART 3BNC117-sensitive plasma viruses	No 3BNC117 resistance mutations found in rebound viruses from sensitive individuals during ATI	3BNC117 at ART starting induces durable, strong HIV-1 Gag-specific CD8^+^ T cell responses
Rosás-Umbert M [32] (2022)	USA	NA	NA	NA	High IFN-γ producers showed better virologic control during ATI Increased HIV-1-specific immunity correlates with partial/complete long-term ART-free control	NA	Higher Gag/Pol CD8^+^ T cell & IFN-γ responses with 3BNC117 + ART than ART alone at 3 and12 months
Cohen YZ [33] (2018)	USA	Open-label/Phase Ib	3BNC117 30 mg/kg: at week 0, 12, 24, and 27; ATI at 2nd days after the w24 infusion	A total of 29 AEs; Three were graded as moderate (chills and myalgia) and one was graded as severe (elevated bilirubin)	3BNC117 doesn’t reduce latent reservoir size Rebound time: a mean of 5.5 weeks (resistant: 3.6 weeks vs. sensitive: 9.2 weeks)	Resistance: polyclonal rebound; sensitivity: restricted diversity Rebound viruses are often recombinants of latent viruses, not the dominant latent reservoir species	NA
Leone PA [34] (2025)	Argentina, United States, Canada, Brazil, Mexico, Peru	RCT, open-label/Phase 2a	N6LS: ~ 4 mg/kg × 1; 40 mg/kg × 1; ~ 1 mg/kg × 1; ~ 10 mg/kg × 1;	All drug-related AEs were grade 1 or 2 The only drug-related AE reported in > 2 participants was headache	An median VL decline: 0.93 (2.60, 0.09) log_10_, ranged from 0.43 log_10_ (~ 1 mg/kg) to 1.72 log_10_ (40 mg/kg)	All drug-related AEs were grade 1 or 2	Median maximum increase in CD4^+^ cell count (cells/mm3): 135 (40 mg/kg), 128 (~ 10 mg/kg), 150 (~ 4 mg/kg), 64 (~ 1 mg/kg); CD4^+^/CD8^+^ ratio: minimal

*RCT* randomized controlled trial, *NA* not available, *ART* antiretroviral therapy, *AEs* adverse effects, *SAEs* serious adverse event, *HD* healthy donor, *PLWH* people living with HIV, *RNA* ribonucleic acid, *DNA* deoxyribonucleic acid, *ATI* analytical treatment interruption, *USA* United States of America, *bNAb* broadly neutralizing antibody, *ADCP* antibody-dependent cellular phagocytosis

**Table 3:** Overview of studies in PLWH receiving combination bNAb therapy

First author (year)	Country	Study design	Schedule	bNAb-related AEs and SAEs	Effectiveness (log _10_ copies/ml)	Resistance	Immune response
Bar-On Y [35] (2018)	USA and Germany	Partial RCT, partial open-label/Phase 1b	3BNC117 and 10–1074: 30 mg/kg × 3 doses: day 0,14 and 28; 10 or 30 mg/kg × 1 dose; placebo × 1 dose	One mild case each of nausea, transaminitis and upper respiratory tract infection	VL reduction: overall: a mean of 1.65 log_10_ copies/ml and stayed low until day 86. Sensitive viruses: a average of 2.05 log_10_, lasting until day 94 Patients with high baseline VL (97,800 copies/ml) rebounded at 8 weeks, while those with low VLs (750 and 2550 copies/ml) maintained suppression for 12 and 16 weeks	In initially sensitive individuals, none of these individuals developed resistance to both antibodies; the rebound viremia was resistant to 10–1074 but remained sensitive to 3BNC117 Rebounding viruses: mutations such as the loss of the N-linked glycosylation site at position 332 (critical for 10–1074 binding) or G471E and N276D mutations, which increase resistance to 3BNC117	No change in CD4^+^ T cell counts
Julg B [36] (2022)	USA	RCT, partial double blind/phase 1b	Part1 × 1 dose (3, 10 and 30 mg/kg): PGDM1400 or placebo; PGDM1400 + PGT121 or placebo; Part2: PGDM1400 + PGT121 (30 mg/kg, each); PGDM1400 + PGT121 + VRC07-523LS (20 mg/kg, each)	One mild case each of oropharyngeal pain and respiratory, thoracic and mediastinal disorders in PGDM1400 group at 3 mg/kg	(Triple bNAbs, *n* = 3): a mean VL reduction of 1.76 log_10_ by day 7, with a nadir of 2.04 log_10_ by day 10 (Combination at 30 mg/kg, *n* = 1): a mean VL reduction of 2.16 log_10_, with a nadir on day 6 post-infusion. VL rebounded by day 30, with ART initiated on day 108	Mutation after bNAb infusion: PGDM1400: the loss of the potential N-linked glycosylation site at residue 160 (losing this glycan site through mutations at either residue 160 and/or 162, such as N160D or N160S and T162A), the presence of glycine at the Env site 166, and a V120I mutation PGT121: the loss of the PNGS at 332, the D325N mutation, shifting a hypervariable V1 PNGS two sites toward the N-terminus relative to other viruses from this participant VRC07-523LS gained a glycan at site 234 and gained a glycan in hypervariable V5 loop	No change in CD4^+^ T cell counts
Sneller MC [37] (2022)	USA	Partial RCT, partial double blind/Phase 1b	3BNC117 and 10–1074 (30 mg/kg, each) or placebo at week 0, 2, 4, 8, 12, 16, 20, 24 and/or AIT at day3 after 1 st infusion Group1: ART was stopped 3 days after the first dose of 3BNC117 + 10–1074, or placebo; group 2: naive PLWH	One case each of rigors, chills, and fever graded as 1–2 in groups 1 and 2	In group 1, 6 of 7 placebo participants rebounded and resumed ART before week 28; none in the bNAb group did In the bNAb group, 2.5 of 7 participants maintained viremia < 40 copies/ml; all placebo participants rebounded within 8 weeks In group 1, viremia lasted a median of 33.4 weeks (35.4 weeks with sensitive virus); 2 had rebounds > 200 copies/ml within 8 weeks with resistant HIV. Placebo arm: 3.4 weeks Group 2: Complete HIV suppression lasted a median of 41.7 weeks (2/5 bNAb-sensitive participants) Combination bNAbs did not have a significant impact on the persistent HIV reservoir	3BNC117 and 10–1074 does not lead to a precipitous accumulation of resistant HIV during long-term virologic suppression, provided that antibody-resistant virus was not present at baseline and serum antibody titers remain high	No significant changes were observed in CD4^+^ T cell counts, CD8^+^ T cell subsets, cytokines (IL-6, IL-8, TNF-α), chemokines (MIP-1ß, RANTES), or immune activation markers (IL-2Rα, CD40, PD-L1, IP-10, CRP, D-dimer) In group 1, levels of polyfunctional HIV Gag-specific CD8^+^ T cells and the breadth of HIV-specific CD8^+^ TCR clonotypes remained stable A modest increase was seen in the depth of HIV-specific CD8^+^ TCR clonotypes following passive bNAb transfer, with a slight expansion in TCR breadth in group 2
Gunst JD [38] (2023)		RCT, double blinded/Phase IIa	Placebo/placebo; Placebo/3BNC117 (30 mg/kg) + 10–1074 (20 mg/kg) at w 0 and 3; both ATI at 1 d before 1 st dose	Eleven were graded mild, two were graded moderate and one was graded severe, with fatigue (*n* = 5) being the most commonly reported AE	Loss of virologic control: Placebo/placebo vs. Placebo/bNAb: at a median of 4.5 w vs. 17 w BNAb administration prevented reservoir expansion during the early phase of the ATI In the placebo/bNAb group, 18% (2 of 11) maintained HIV RNA < 20 copies/ml throughout the 25‑week ATI	As 3BNC117 declined, most bNAb group participants rebounded on 10–1074 monotherapy In BNAb-treated individuals, 10–1074 resistance rose from 13% (screening) to 89% (rebound) Resistant proviruses caused early viral rebound in some participants	HIV-1 CD4^+^ T cell frequency remains stable across all groups BNAb recipients with < 50 copies HIV RNA showed no major CD8^+^ T cell response increase BNAb-treated individuals with HIV RNA > 50 copies had higher CD8^+^ T cell responses at week 13 vs pre-ATI
Mendoza P [39] (2018)	USA and Germany	Open-lable/Phase 1b	3BNC117 + 10–1074 (30 mg/kg, each) at weeks 0, 3 and 6, and ATI 2 days after the 1 st infusion	Two mild malaise/fatigue	Rebound time: in patients with antibody-sensitive latent viral reservoirs: 9/11 > 15 weeks (median 21 weeks); 2/11 rebounded at weeks 5 and 7 (pre-existing resistance to one of the two bNAbs) The antibodies restricted the outgrowth of latent viruses in vivo	Rebound viruses exhibited resistance mutations to 10–1074 but remained sensitive to 3BNC117; no cases of double resistance were observed in 9 individuals	Mean CD4^+^ T cell counts were 685 at first infusion and 559 at rebound
Niessl J [40] (2020)	USA and Germany	Open- lable/Phase 1b	3BNC117 + 10–1074 (30 mg/kg, each) at 0, 3 and 6 weeks, and ATI at 2 days after1st bNAbs (*n* = 9); ART only (*n* = 13)	NA	Rebound time: for at least 15 weeks after ATI (viruses sensitive to both bNAbs)	NA	Several subsets and response of Gag-specific mono- or polyfunctional CD8^+^ and CD4^+^ T cells were augmented at weeks 6/7 and 12 compared to baseline for bNAb-treated individuals CD8^+^ T cell responses decreased by week 12 but remained elevated compared to baseline for IFN-γ, TNF-α, and MIP1-β Total cytokine^+^ CD4^+^ T cells remained above baseline at all time point
Shapiro RL [41] (2023)	Botswana	Open- label/Phase I/II	ART started from day 7 after birth and continued ≥ 96Ws, and alongside bNAb ≥ 8Ws and bNAb ≥ 24Ws ( 10–1074 30 mg/kg q4w; VRC01LS 30 mg/kg × 1 dose and 15 mg/kg q4w)	One grade 3 (neutropenia)	44% vs 40% patients: HIV-1 RNA < 400 and < 40 copies/ml for 24 weeks 56% patients: Viremia ≥ 400 copies/ml at a median of 4 weeks	Some degree of reduced neutralization by 10–1074 or VRC01LS	No child in either group showed a concerning CD4 decline during the study
Niesar A [42] (2026)	Botswana	NA	ART started from day 7 after birth and continued ≥ 96Ws, and alongside bNAb ≥ 8Ws and bNAb ≥ 24Ws ( 10–1074 30 mg/kg q4w; VRC01LS 30 mg/kg × 1 dose and 15 mg/kg q4w)	NA	NA	No experimental evidence supporting a decrease of viral reservoir cells during bnAb therapy	Higher frequencies of NKG2A-expressing NK cells are linked to delayed viral rebound kinetics during bnAb-only therapy No statistically significant differences of the frequencies of monocytes and their subtypes as well as dendritic cells at the start of bnAb-only treatment or at ART reinitiation No evidence for an expansion or increase of HIV-1–specific CD4^+^ or CD8^+^ T cell responses during bnAb treatment
Julg B [43] (2024)	USA	Open- label/Phase 1/2a	Group 1A: PGT121 + VRC07-523LS: (30 mg/kg, each) × 1 dose; Group 1B: PGT121 + PGDM1400 + VRC07-523LS: (20 mg/kg, each) × 1 dose; Group 2: PGT121 + PGDM1400 + VRC07-523LS:20 mg/kg × 3–6 doses qm, and ATI at 2 days after the initial infusion	One grade-1 dizziness in PGT121 + VRC07-523LS; One grade-1 presyncope, injection site swelling, AST increased and night sweats in PGT121 + VRC07-523LS + PGDM1400	Viral rebound for at least 28 weeks: 10/12 (83%); for at least 38–44 weeks: 5/12 (42%) Two rebounds occurred at week 6 and week 10 due to baseline drug resistance BNAb infusions did not significantly reduce the peripheral HIV-1 reservoir	Most rebound viruses were resistant to PGT121 and PGDM1400 but remained sensitive to VRC07-523LS Resistance to PGT121 involves loss of the 332 glycan, or H330Y and G324R mutations in the 324GDIR327 contact site Resistance to PGDM1400 occurs through mutations N130 and V169 or K168E and R190S	BNAb infusion linked to immune activation, T cell exhaustion, and inflammation
Gaebler C [44] (2022)	USA	Open- lable/phase 1b	Group1: 3BNC117 and 10–1074 (30 mg/kg, each) at w 0, 2, 4, 8, 12, 16, 20 and ATI at day 2 after 1 st infusion or group 2 at week 26 after 1 st infusion in group2 Group3: ART alone	Twenty‑eight percent (25 of 90) experienced grade‑1 AEs, including headache, infusion‑site ecchymosis/hematoma or edema, malaise/fatigue, upper‑respiratory infection symptoms, dizziness, fever/chills, flatulence, flushing, light‑headedness, ocular irritation, pyrosis, and sinusitis	Group 1: 76% (13/17) viral rebound ≥ 20 weeks after ATI. Rebound at a median of 28.5 weeks, following repeated infusion; rebound at a median of 32 weeks, after receiving 7 doses of bNAb; 2 cases experienced rebound at 48 weeks; 1 case remained suppressed for 2 years Group 2: Rebound: at meidan of 7 weeks after ATI Repeated bNAbs alter intact latent reservoir size/composition over 6 months in virologically suppressed individuals	The mutations observed at the target site of 10–1074	CD4^+^ count: 729 cells/μl (start of treatment), 660 cells/μl (viral rebound), 1 cases: a more than 30% decline (group1) No significant changes in activation markers expressed by CD4^+^ or CD8^+^ T cells

*q4w* once every four weeks, *W* week, *RCT* randomized controlled trial, *NA* not available, *ART* antiretroviral therapy, *AEs* adverse effects, *SAEs* Serious Adverse Event, *HD* healthy donor, *RNA* ribonucleic acid, *ATI* analytical treatment interruption, *USA* United States of America, *PLWH* people living with HIV, *RNA* ribonucleic acid

These studies were conducted across several geographic settings, with most (*n* = 18) performed exclusively in the United States. The remaining trials were conducted in the United States and Switzerland (*n *= 1), Thailand (*n* = 2), Botswana (*n* = 2), Denmark and England (*n* = 1), the United States and Germany (*n* = 5), and across Argentina, the United States, Canada, Brazil, Mexico, and Peru (*n* = 1) (Tables 1, 2, 3). Among the included studies, 9 were Phase Ia trials, 14 were Phase Ib, 3 were Phase I/II, and 4 were Phase II.

A total of 10 bNAbs were evaluated, including VRC01, VRC01LS, VRC07-523LS, PGT121, PGDM1400, 10–1074, PGT121.414LS, N6LS, PGDM1400LS, and 3BNC117. These antibodies were administered as monotherapy or in dual- or triple-combinations regimens. In total, 30 distinct bNAb treatment regimens were identified, including 11 trials evaluating combination‑antibody strategies (Tables 1, 2, 3). Across all included studies, 801 participants were included, comprising 340 HIV-1 negative individuals, 300 aviremic PLWH receiving suppressive ART, and 161 viremic PLWH who were either off ART or ART-naïve. Detailed study and participant characteristics are presented in Table 1 and Additional files 4–5: Tables S4–S5.

### Pharmacokinetic profile

#### Half-life (t_1/2_) of bNAbs

**Fig. 2:**
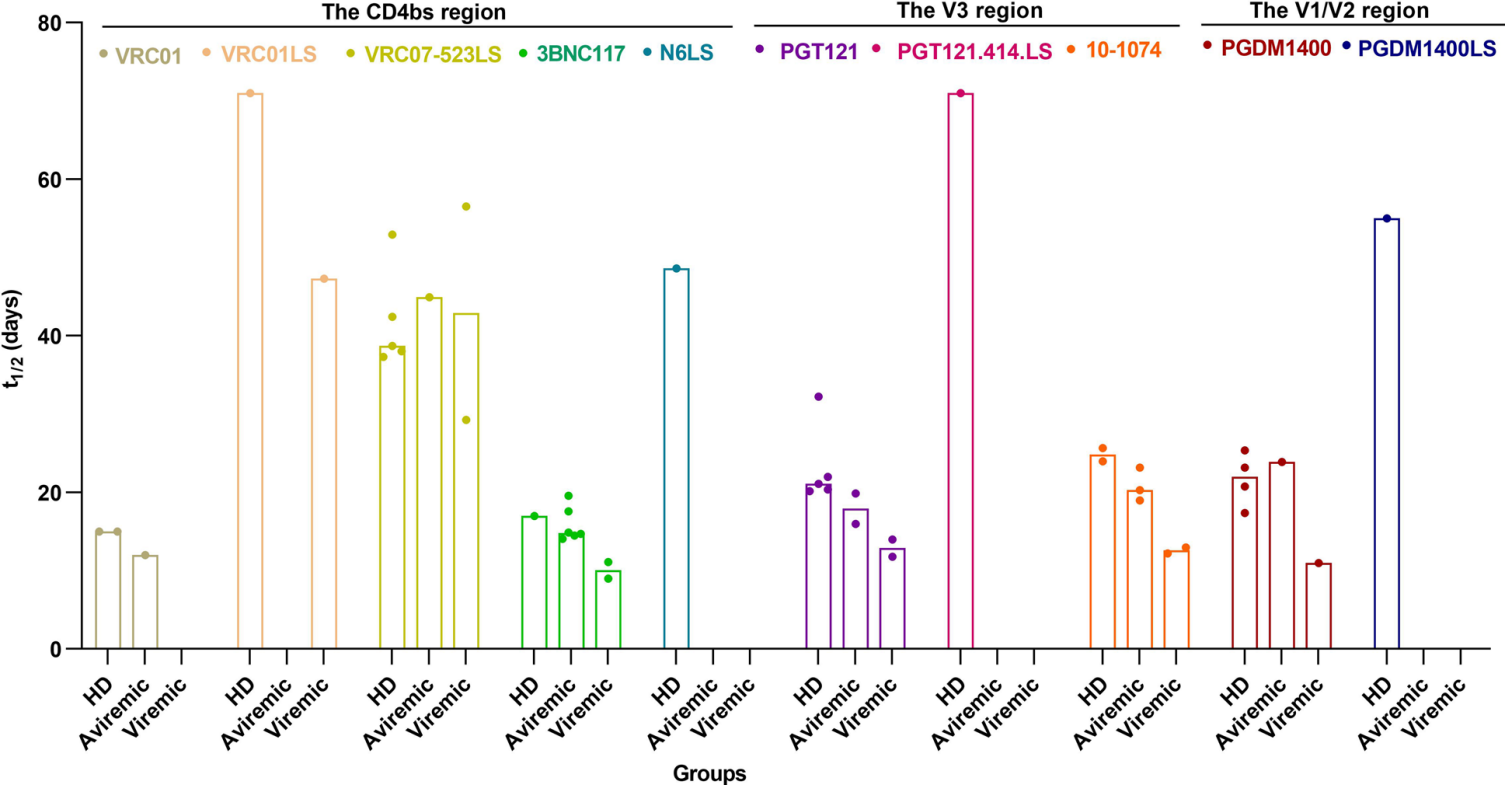
Half-life of bNAbs in HIV-1-negative individuals and PLWH. Each point represents the mean or median half‑life (t_1/2_) of the corresponding bNAb reported in an individual clinical trial. *PLWH* people living with HIV-1, *HD* healthy individuals, *bNAbs* broadly neutralizing antibodies. Viremic and aviremic participants are indicated separately

Among the 34 included trials, 22 reported the t_1/2_ of bNAbs administered individually or in combination in HIV-1-negative individuals and PLWH. Two consistent patterns were observed. First, LS-modified bNAbs generally showed longer half-lives than their parental antibodies. Second, half-life tended to be shorter in PLWH with detectable viremia than in aviremic PLWH or HIV-1-negative individuals (Additional file 6: Table S6 and Fig. 2).

Among CD4bs-targeting antibodies, VRC01 had a t_1/2_ of approximately 15 days in HIV-1-negative individuals and 12 days in aviremic PLWH [11]. Its LS-modified variant, VRC01LS, extended the half-life to 71 days in HIV-negative individuals, representing an approximately 4.7-fold increase over VRC01, although this declined to 47.3 days in viremic PLWH [13, 25]. Similarly, VRC07-523LS showed a t_1/2_ ranging from 37.7 to 52.9 days in HIV-negative individuals, decreasing to 29.3 days in viremic PLWH and rising again to 44.9 days in aviremic PLWH [14, 17]. 3BNC117 exhibited a t_1/2_ of 17 days in HIV-negative individuals, which declined to 9–11.1 days in viremic PLWH, while remaining comparable in aviremic PLWH (14.1–19.6 days) [20, 27, 33, 34, 42]. N6LS also showed a relatively long half‑life of 48.6 days in HIV‑negative individuals, compared with 9.9–24.1 days in viremic PLWH across different dosing regimens [18, 34].

Among V3-glycan targeting antibodies, PGT121 had a t_1/2_ of 20.2–32.2 days in HIV-negative individuals, decreasing to 16–19.9 days in aviremic PLWH and 11.8–14 days in viremic PLWH [15, 22, 36, 43]. Its LS variant, PGT121.414LS, extended the half-life to 71 days in HIV-negative individuals, corresponding to a 2.2- to 3.5-fold increase over the parental antibody. By comparison, 10–1074 displayed a t_1/2_ of 24–25.7 days in HIV-negative individuals, 12.2–23.2 days in aviremic PLWH, and 13–19 days in viremic PLWH [15, 23, 34, 39, 44].

For V1/V2-targeting antibodies, PGDM1400LS demonstrated a substantially prolonged t_1/2_ of 55 days in HIV-negative individuals, approximately 2- to threefold longer than that of the parental PGDM1400 (17.4–25.4 days). In PLWH, the half-life of PGDM1400 was 23.9 days in aviremic PLWH and decreased to 11 days in viremic PLWH [15, 36, 43].

#### Plasma concentration of bNAbs

**Fig.3:**
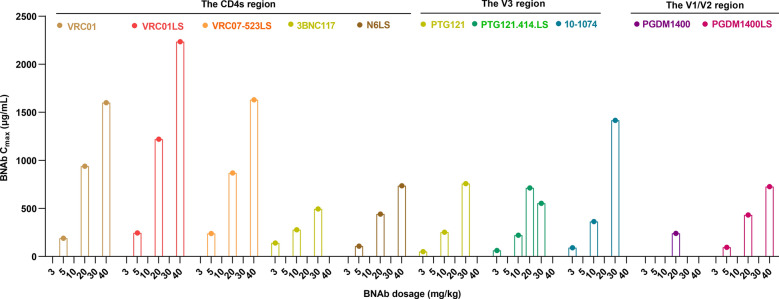
C_max_ characteristics of bNAbs in HIV-1-negetive individuals and PLWH. Each point represents the mean or median C_max_ (µg/ml) of the corresponding bNAb at a given dose, as reported in an individual clinical trial. *PLWH* people living with HIV-1, *bNAbs* broadly neutralizing antibodies, *C*_*max*_ peak plasma concentrations

Peak plasma concentrations (Cmax) of bNAbs were reported in 15 trials involving both HIV-negative individuals and PLWH following individual or combination intravenous administration (Additional file 6: Table S6 and Fig. 3). Overall, Cmax increased with dose across antibodies after single infusions.

For example, at a dose of 5 mg/kg, VRC01, VRC01LS, and VRC07-523LS reached Cmax values of 210, 246, and 240 μg/ml, respectively, in HIV-1 negative individuals (Additional file 6: Table S6). At 20 mg/kg, these values increased to 1100, 1221, and 869 μg/ml, respectively. At the highest tested dose of 40 mg/kg, the corresponding Cmax values were 1500 μg/ml for VRC01, 2234 μg/ml for VRC01LS, and 1630 μg/ml for VRC07-523LS. Overall, LS-modified antibodies generally achieved higher Cmax values than their parental counterparts, with VRC01LS reaching the highest levels among the VRC01-based antibodies [11, 13, 14] (Additional file 6: Table S6 and Fig. 3).

Similarly, PGDM1400LS achieved a Cmax of 432.6 μg/ml at 20 mg/kg, compared with 241.4 μg/ml for the unmodified PGDM1400 [15, 19]. An exception was observed for PGT121.414LS, which showed Cmax values that were similar to, or slightly lower than, those of PGT121 in PLWH [16, 22], possibly due to differences in blood sampling time points.

Beyond peak concentrations, LS-modified antibodies such as VRC01LS and VRC07-523LS also maintained substantially higher plasma concentrations over time (Additional file 6: Table S6 and Additional file 7: Fig. S1). At Week 4 post-infusion at 40 mg/kg, VRC01LS and VRC07-523LS achieved plasma concentrations of 651 and 274 μg/ml, respectively, compared with 89 μg/ml for VRC01 [11–14]. This pattern persisted through week 12, with VRC01LS maintaining the highest concentration at 326 μg/ml, followed by VRC07-523LS at 85 μg/ml and N6LS at 81 μg/ml (Additional file 6: Table S6 and Additional file 7: Fig. S1) [11–14].

Overall, LS modification increased Cmax and prolonged half-life relative to parental antibodies, generally by approximately 1.4- to 1.8-fold and 2- to 5-fold, respectively. The prolonged half-life supported extension of dosing intervals from weekly or every 2 week administration to every 4 to 12 weeks. Although viremia reduced the absolute concentrations of both parental and LS-modified antibodies, the reduction was generally smaller for LS-modified antibodies, at approximately 20%, than for their parental counterparts, for which reductions were typically around 30 to 40%.

### ADA development and IgG GM allotyping

ADA responses were evaluated in 20 of the 34 clinical trials (Table 1 and Additional files 4–5: Tables S4–S5). Across these studies, the development of ADA against administered bNAbs was rare. In one trial, only two of eight participants developed detectable ADAs [22]. In another trial, 18 of 46 participants had low ADA titers, but only two remained ADA-positive at low levels at the end of follow-up [34]. No ADA responses were observed in any of the remaining bNAb trials [11–20, 24–26, 28, 29]. These findings indicate that the immunogenicity of bNAbs is generally low and that rare ADA development has little or no apparent impact on safety, pharmacokinetics, or antiviral activity. Additionally, IgG1 GM allotyping was not significantly associated with the pharmacokinetic profiles of VRC01, VRC01LS, or VRC07-523LS [11, 13, 14] (Table 1), suggesting that host allotype differences do not substantially influence bNAb metabolism or clearance.

### Impact of bNAbs on HIV-1 suppression and latent reservoir dynamics

#### Neutralizing activity of bNAbs in healthy adults

Eight of the nine trials conducted in HIV-negative individuals demonstrated that both single-agent and combination administration of nine bNAbs retained broad and potent neutralizing activity against HIV-1 [11–19] (Table 1). The antibodies evaluated included VRC01, VRC01LS, VRC07-523LS, N6LS, PTG121, PGT121.414LS, 10–1074, PGDM1400, and PGDM1400LS [11–19]. For instance, VRC01 effectively neutralized HIV-1 subtypes A, B, and C following subcutaneous or intravenous administration. In vitro, it exhibited low IC80 values ranging from 0.09 to 1.6 μg/ml. When administered intravenously at doses of 10–30 mg/kg, VRC01 neutralized 93–94% of clade B and 75–84% of clade C tier 2 pseudoviruses at 24 weeks, as measured by IC50 [11]. Notably, this breadth of coverage remained substantial even under the more stringent IC80 criterion, with 82–93% of clade B and 58–75% of clade C strains neutralized at trough serum concentrations [11].

Similarly, VRC07-523LS exhibited neutralization breadth and potency comparable to, or greater than, those of VRC01 or VRC01LS despite lower circulating concentrations [14]. N6LS, administered as a single intravenous dose of 40 mg/kg, maintained activity against approximately 80% of viral strains from clade C and multiclade panels for up to three months [18]. Compared with VRC01, N6LS showed greater potency while maintaining neutralization breadth similar to that of VRC07-523LS [18]. PGDM1400LS showed activity comparable to that of its parental antibody, PGDM1400, and both demonstrated greater potency and breadth against circulating clade C viruses than against clade B viruses [19].

Notably, the dual combination of PGT121.414.LS and VRC07-523LS demonstrated superior efficacy compared with PGT121.414.LS monotherapy. This effect was further enhanced with the triple combination of PGDM1400, PGT121, and VRC07-523LS, which achieved the broadest neutralization coverage among all tested regimens and outperformed dual combinations such as PGT121 + VRC07-523LS, PGDM1400 + VRC07-523LS, and 10–1074 + VRC07-523LS [15, 16]. These findings underscore the synergistic potential of bNAb combinations to achieve greater neutralization breadth and potency than single agents, likely due to complementary epitope targeting.

#### Impact of bNAbs on HIV-1 suppression in PLWH

**Fig. 4:**
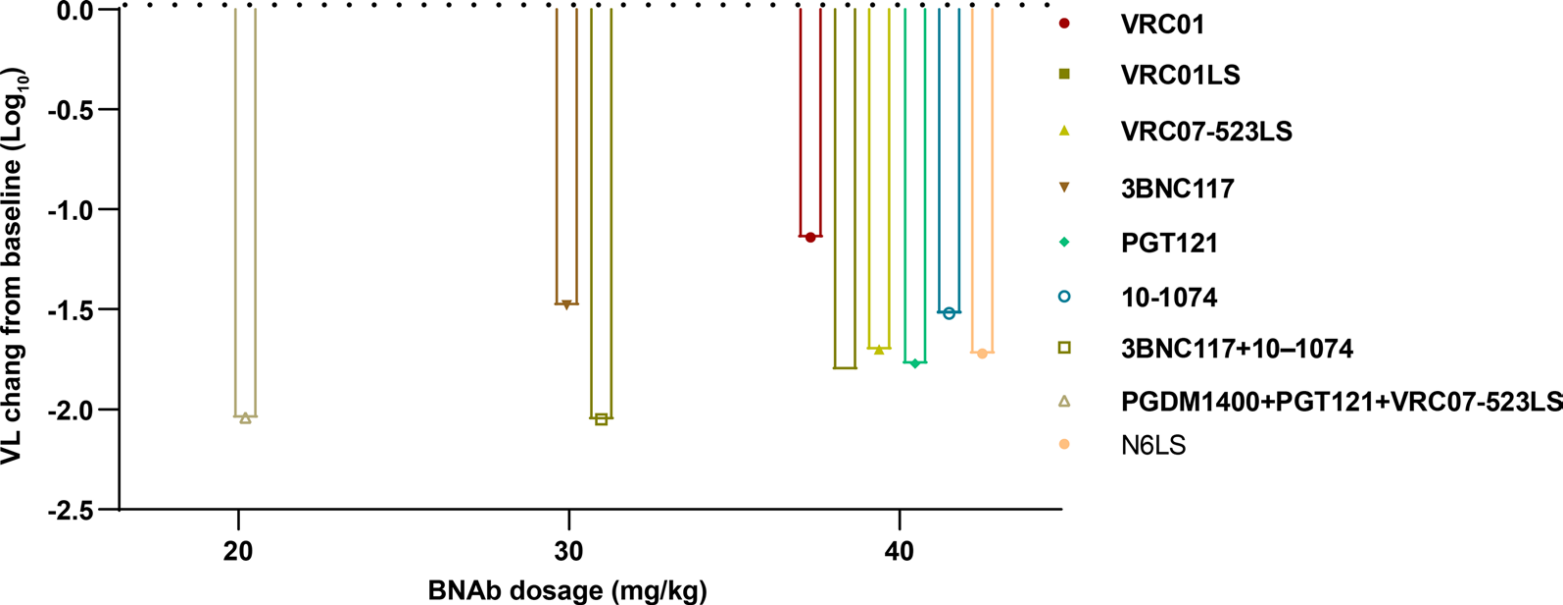
Viral load reduction from baseline after bNAb administration. Each point represents the mean or median change in viral load (VL) from baseline after administration of the corresponding bNAb at a given dose, as reported in an individual clinical trial. *bNAbs* broadly neutralizing antibodies, *VL* viral load

**Fig. 5:**
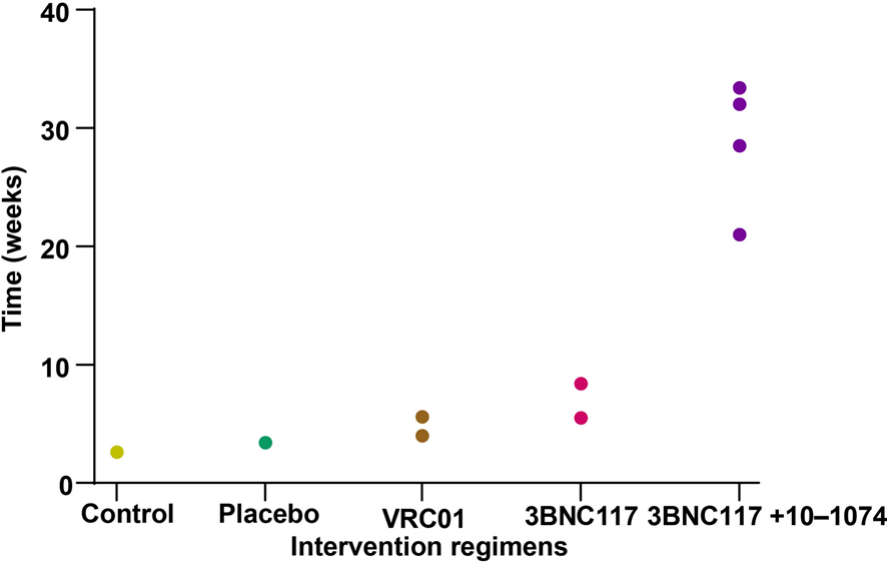
Time to viral rebound after ATI following bNAb administration. Each point represents the mean or median time to viral rebound (weeks) after ATI following bNAb administration, as reported in an individual clinical trial. *bNAbs* broadly neutralizing antibodies, *ATI* analytical treatment interruption

BNAbs have been evaluated across four key dimensions of HIV-1 virologic control: (1) complete viral suppression (3 of 34 trials), (2) direct plasma VL reduction in viremic individuals (10 of 34 trials), (3) delayed viral rebound after ATI (8 of 34 trials), and (4) prolonged time to loss of virologic control (2 of 34 trials) (Tables 2 and 3; Figs. 4, 5).

In viremic PLWH harboring bNAb-sensitive virus, single-agent bNAbs generally produced modest VL reductions followed by rapid rebound (Table 2 and Fig. 4). At doses of 30–40 mg/kg, VRC01, VRC01LS, VRC07-523LS, 3BNC117, 10–1074, PGT121, and N6LS achieved VL declines of 0.93–1.8 log_10_, typically observed shortly after infusion and returning toward baseline in most participants by day 28 [20–25, 34]. Lower doses, such as 1–3 mg/kg of 3BNC117 and N6LS, produced only transient or modest reductions (Table 2) [20, 34].

In contrast, dual- and triple-bNAb combinations produced more profound and durable virologic effects (Table 3 and Fig. 4). The combination of 3BNC117 and 10–1074 reduced VL by a mean of 2.05 log₁₀ copies/ml in PLWH with sensitive viruses, with suppression lasting up to 94 days [35]. Similarly, a triple regimen comprising PGDM1400, PGT121, and VRC07-523LS achieved reductions of up to 2.04 log₁₀ copies/ml across all participants [36] (Table 3). These data suggest that combination bNAbs can produce additive or synergistic effects, likely because of broader neutralization coverage and a reduced risk of viral escape.

Complete viral suppression during ATI was also observed more consistently with combination strategies. Whereas VRC01 monotherapy extended the time to HIV-1 RNA rebound to 29 days compared with 14 days in the placebo group [29], the combination of 3BNC117 and 10–1074 achieved viral suppression for at least 8 weeks in 5 of 7 participants, with two individuals maintaining undetectable viremia for over 40 weeks [37]. Notably, the triple-bNAb combination maintained suppression in 83% of participants for at least 28 weeks and in 42% for 38–44 weeks after ATI [37] (Table 3). Although single-agent bNAbs generally produced short-lived VL declines, rare cases of prolonged complete suppression were also observed; for example, two individuals receiving PGT121 maintained VLs below 40 copies/ml for over 168 days [22, 24] (Table 2).

Similar trends were observed for time to viral rebound (Tables 2, 3; Fig. 5). VRC01 monotherapy resulted in a mean time to viral rebound of approximately 4–5.6 weeks [28] (Table 2). For 3BNC117, the mean time to viral rebound ranged from 5.5 to 8.4 weeks overall, but varied markedly according to baseline viral sensitivity and dosing regimen, with longer rebound times observed in individuals harboring sensitive virus than in those with resistant virus (9.2 vs 3.6 weeks), and after three infusions than after two (9.9 vs 6.7 weeks) [27, 33] (Tables 2, 3). By contrast, the 3BNC117 + 10–1074 regimen significantly delayed rebound, with median times exceeding 15–33 weeks [37, 44] and reaching 35.4 weeks in individuals harboring sensitive viruses [37, 39, 40]. Furthermore, 76% of participants remained suppressed for at least 20 weeks [44] (Table 3), and one case maintained suppression for more than two years. Longer delays in rebound were associated with earlier ART initiation, baseline viral sensitivity of bNAbs, and a greater number of infusions.

Time to loss of virologic control was assessed in two trials. VRC01 monotherapy delayed loss of virologic control to a median of 33 days, compared with 14 days in the placebo group [29] (Table 2). In contrast, 3BNC117 + 10–1074 prolonged virologic control to a median of 17 weeks, compared with 4.5 weeks in the placebo group, again highlighting the superiority of combination therapy [38] (Table 3).

Overall, combination bNAb regimens produced greater and more sustained virologic effects than single agents, including deeper viral load reductions, longer time to rebound, higher rates of suppression during ATI, and delayed loss of virologic control. Outcomes varied according to bNAb combinations, dosing schedules, baseline viral sensitivity, and participant characteristics.

#### Effect of bNAbs on latent reservoir dynamics

Ten studies evaluated the effects of bNAbs on the HIV-1 latent reservoir (Tables 2, 3). Five of these studies, including those investigating single infusions of VRC01 or 3BNC117 and combination regimens such as 3BNC117 plus 10–1074 or PGDM1400/PGT121/VRC07-523LS, reported no measurable reductions in total HIV-1 reservoir size among individuals receiving ART [24, 26, 33, 37, 43].

In contrast, five studies suggested that bNAbs may affect reservoir dynamics under specific conditions. In individuals harboring bNAb-sensitive viruses, 3BNC117 monotherapy was associated with restricted viral outgrowth limited to low-diversity lineages [27]. Early administration of 3BNC117 during ART initiation reduced the frequency of CD4⁺ T cells harboring intact HIV-1 proviruses [31]. Moreover, in ART-suppressed individuals undergoing ATI, the combination of 3BNC117 and 10–1074 prevented reservoir expansion and restricted viral rebound to narrow phylogenetic clusters [39]. Repeated infusions of the same combination over six months further reduced the size of the intact reservoir and altered its clonal composition [38, 44]. Together, these findings suggest that although bNAbs may not consistently reduce total reservoir size, they may influence reservoir dynamics in specific settings, particularly when initiated early, used in combination, and matched to baseline viral sensitivity.

### Resistance to bNAbs

#### Mutation-driven resistance to individual bNAbs

Escape viruses frequently harbor mutations involving amino acid substitutions near antibody binding sites (Tables 2, 3). For VRC01, resistance-associated mutations occur mainly in the V5 and CD4-binding loops, particularly around β20/β21, loop D, and positions 280, 429, and 462, as well as through alterations in V5 loop length [24]. Resistance to VRC07-523LS has been associated with the G459D mutation [36]. By contrast, PGDM1400 resistance appears to arise through multiple mechanisms, including substitutions at N130, K168E, or R190, mutations at residues 161 and 165, and structural destabilization of the V1 loop and loop19 region due to loss of a paired cysteine residue [41]. For 3BNC117 and 10–1074, resistance commonly involves amino acid changes in or near their Env contact residues—positions 274–283, 364–374, 455–471, 332, and 363 for 3BNC117, and positions 324–327 and 332–334 for 10–1074—with some V5 loop changes causing steric hindrance to 3BNC117 binding [20, 27, 35, 37–39, 44]. Resistance to PGT121 has been linked to mutations in the V1 loop, particularly at positions 330–335 [22].

#### Cross-resistance patterns observed with different bNAbs

Cross-resistance patterns have been observed, particularly among bNAbs targeting overlapping Env regions (Tables 2, 3). VRC01 treatment generally does not induce Env mutations that alter susceptibility to other bNAbs, including 3BNC117, 10–1074, PGT121, and UB-421, with only minimal differences observed between pre-ART and rebound isolates [28]. Although 3BNC117 treatment rarely increases resistance to 10–1074, rebound viruses are often recombinants derived from diverse latent reservoirs. Resistant latent strains tend to cause polyclonal viral rebound, whereas sensitive strains are associated with more limited viral diversity [27, 33].

PGT121 and 10–1074 exhibit marked cross-resistance: most PGT121-resistant viruses are also resistant to 10–1074, and vice versa [22, 23]. However, these V3-targeting bNAb-resistant viruses frequently remain sensitive to bNAbs directed against other Env regions, such as PGDM1400 and 3BNC117 [22]. Importantly, no correlation has been observed between the emergence of 10–1074 resistance and resistance to non-V3-targeting bNAbs, including VRC01, 3BNC117, and PGDM1400 [23]. Together, these findings support the rationale for combining bNAbs that target non-overlapping epitopes in order to reduce the risk of viral escape and enhance antiviral activity.

#### Resistance emergence during and after treatment interruption

Of the 34 studies, 9 reported bNAb-driven selection pressure during ATI (Tables 2, 3). In two studies involving early-ART, one evaluating VRC01 and the other 3BNC117, no resistance mutations were detected in rebound viruses [29–31]. In the VRC01 study, the mean trough concentration at the time of viral rebound was 221 μg/ml, approximately 50-fold higher than the in vitro IC80 [29, 30], suggesting that rebound was not simply attributable to subtherapeutic antibody exposure. In the 3BNC117 study, early concurrent ART likely limited viral replication and thereby reduced the opportunity for selection of resistant variants [31]. During the subsequent ATI phase, plasma 3BNC117 concentrations were not directly measured; however, based on the known half-life of the antibody, serum levels were likely substantially reduced by day 400, which may also have limited selective pressure for resistance emergence. Nevertheless, nearly all bNAbs, whether administered individually or in combination, appeared to exert selective pressure on rebounding virus populations.

For the 3BNC117 + 10–1074 combination, one study reported no resistance mutations to either antibody [37], whereas four others identified emerging resistance to 10–1074 during viral rebound [35, 38, 39, 44]. Resistance often emerged when serum antibody levels fell below putative protective thresholds, namely < 10 μg/ml for both 10–1074 and 3BNC117 [35, 39, 44]. In contrast, one study reported viral rebound despite persistently elevated serum concentrations (73.3 μg/ml for 10–1074 and 13.2 μg/ml for 3BNC117) [38]. These findings suggest functional monotherapy may occur during later phases of treatment. In such cases, rebound was associated with a marked increase in the frequency of 10–1074 resistance, from 13 to 89% [37, 38].

For the triple combination of PGT121, PGDM1400, and VRC07-523LS, viral rebound occurred when PGT121 and PGDM1400 concentrations fell below 10 μg/ml and VRC07-523LS concentrations fell below 100 μg/ml, often accompanied by resistance to PGT121 and PGDM1400 but preserved sensitivity to VRC07-523LS [43]. In the same study, however, one participant (ID 65021) experienced early viral rebound despite maintaining VRC07-523LS serum concentrations above 100 μg/ml and without detectable VRC07-523LS resistance mutations; the rebounding virus was resistant to PGT121 and PGDM1400. This finding suggests that preserved plasma exposure and in vitro sensitivity to VRC07-523LS alone may not always be sufficient to ensure durable viral suppression [43]. Possible explanations include incomplete antibody penetration into tissue reservoirs, limited clearance of infected cells despite neutralization of free virions, or transient viral reactivation from persistent reservoirs. Notably, early rebound in one participant was also linked to resistance to VRC07-523LS, suggesting that this antibody may itself drive escape under certain conditions. Together, these findings emphasize the importance of maintaining adequate serum concentrations and selecting patients on the basis of baseline viral sensitivity [36].

### Effects of bNAbs on immune responses

Most studies assessing the immunologic effects of bNAbs reported no significant changes in overall immune parameters (Tables 2 and 3). In 10 studies, bNAbs administration was associated with stable CD4⁺ and/or CD8⁺ T-cell counts. Similarly, three trials found largely unchanged levels of circulating cytokines, chemokines, markers of immune activation, and markers of T-cell activation, including the frequency of polyfunctional HIV Gag-specific CD8⁺ T cells and the diversity of HIV-specific CD8⁺ T-cell receptor clonotypes [22, 37, 42, 44]. These findings suggest that a vaccinal effect was not consistently observed across studies. Instead, modulation of innate and cellular immunity after bNAb infusion may also have contributed to the prolonged virologic control observed even after antibody levels declined to low or undetectable levels [36].

Conversely, six studies reported evidence of enhanced B- and/or T-cell responses following bNAb therapy, particularly in the settings of early intervention or ATI. In individuals newly diagnosed with HIV-1, early administration of 3BNC117 in combination with ART significantly enhanced Pol- and Gag-specific CD8⁺ T-cell responses, as well as Gag-induced interferon-γ production after ATI [31, 32]. Additionally, the combination of 3BNC117 and 10–1074 enhanced both CD8⁺ and CD4⁺ Gag-specific T-cell responses and was associated with the emergence and expansion of novel T-cell clonotypes during ATI [39, 40]. These findings were further supported by another trial showing increased CD8⁺ T-cell responses among participants with detectable HIV-1 viremia (> 50 copies/ml) following ATI [38]. Furthermore, 3BNC117 was shown to elicit HIV-specific immune responses in viremic PLWH regardless of baseline antibody sensitivity [21]. Taking together, these observations are consistent with a possible vaccinal effect in some viremic or early-treated settings.

Overall, although most studies reported relative immunologic stability following bNAb administration, select trials demonstrated enhanced HIV-specific T-cell responses under conditions of early intervention, viremia, or ATI. These findings suggest that a vaccinal effect may occur under specific circumstances, although it is not consistently observed. In addition, modulation of innate and cellular immunity may also contribute to virologic control after bNAb levels wane.

### Safety

#### Overall safety and common adverse events (AEs)

Across the 34 included trials, intravenous administration of bNAbs at doses ranging from 1 to 40 mg/kg, whether as single agents or in combination, was generally well tolerated (Additional files 8–10: Tables S7–S9). Only 0.4% of participants (3/801) discontinued bNAb infusion due to AEs: one VRC01 infusion was stopped because of a transient rash [26], one participant experienced fever and rigors during the initial 3BNC117 infusion and therefore did not receive subsequent doses [37], and another discontinued 3BNC117 + 10–1074 therapy because of a decline in CD4⁺ T-cell count, which prompted ART resumption [44].

Most AEs were mild and self-limited. Commonly reported events included local injection-site reactions, such as pain, tenderness, and bruising, as well as systemic symptoms including headache, fatigue, myalgia, chills, and gastrointestinal discomfort (e.g., nausea and vomiting) (Additional files 8–10: Tables S7–S9). Upper respiratory tract infections were also frequently observed, particularly in trials involving 10–1074 alone or in combination with VRC07-523LS, PGDM1400, or PGT121 [15, 22, 36, 43]. Reductions in platelet count were noted in some combination regimens, although these events were typically asymptomatic and resolved spontaneously [43].

Importantly, neither the frequency nor the severity of local or systemic AEs appeared to increase significantly with higher doses, repeated infusions, or the use of dual or triple- bNAb combinations [14, 15, 22, 27, 36].

#### Other bNAb-related AEs

In addition to local and systemic symptoms, bNAb-associated AEs also included a range of mild to moderate clinical and laboratory abnormalities (Tables 1, 2, 3). Across trials, the reported frequency of these events ranged from 1% to 19.2% and included elevated liver enzymes, hyperbilirubinemia, creatinine fluctuations, neutropenia, nervous system symptoms (e.g., dizziness, paresthesia, and headache), infections, gastrointestinal complaints, and diaphoresis (Tables 1, 2, 3) [11–16, 18–20, 22, 25, 26, 28, 29, 31, 33–39, 41, 43, 44].

Some AEs appeared to be antibody-specific. VRC01 was associated with one case each of varicella‑zoster virus reactivation and severe generalized urticaria (Tables 1, 2) [12, 29]. N6LS was linked to moderate neutropenia and mild alanine aminotransferase elevation (Table 1) [18]. VRC07‑523LS caused mild paresthesia (1/9, 11%) and transient neutropenia (1/9, 11%) (Table 2) [25, 33], whereas VRC01LS showed a single case of grade 3 neutropenia (Table 3) [41]. PGDM1400 was associated with mild respiratory, thoracic, and mediastinal disorders (grade 1) (Table 3) [36]. In addition, conjunctival erythema was reported in two participants receiving 3BNC117 (3.4%) (Table 2) [27], and one case of severe bilirubin elevation was also observed (Table 2) [33]. Finally, dry‑eye symptoms were reported in 3% of participants receiving 10‑1074 (Table 2) [23].

Combination regimens were associated with a broader spectrum of AEs. For example, 3BNC117 + 10–1074 was linked to hyperhidrosis, hypotension, and infusion site ecchymosis or hematoma in up to 19.2% of participants, as well as upper respiratory symptoms, lightheadedness, ocular irritation, pyrosis, and sinusitis (each occurring in 3.85%) [37, 42]. Mild bilateral hand paresthesia was observed with 10–1074 + VRC07-523LS [15], whereas 10–1074 + VRC01LS was accompanied by laboratory abnormalities including anemia (10%), hyponatremia (3%), low glucose (5%), and hyperglycemia (2%) [41].

## Discussion

This systematic review provides the most comprehensive synthesis to date of clinical trials evaluating intravenously administered bNAbs in both HIV-1-negative individuals and PLWH. By collating and critically appraising data from 34 trials involving 10 distinct bNAbs across monotherapy, dual-combination, and triple-combination regimens, we provided an integrated assessment of their pharmacokinetic characteristics, antiviral efficacy, resistance patterns, immunologic effects, and safety profiles. Our findings highlight three key themes: (1) LS modification significantly prolongs antibody half-life, although this advantage is attenuated by viremia; (2) combination bNAb regimens achieve deeper and more durable viral suppression, delay rebound, and reduce resistance emergence compared with monotherapy; and (3) bNAbs exhibit low immunogenicity and a favorable safety profile. Together, these insights offer a foundation for the design of next-generation antibody-based strategies aimed at durable HIV-1 remission and functional cure.

Durable viral suppression and delayed rebound were observed most consistently with dual- or triple-bNAb regimens [35–44], whereas monotherapy typically led to viral rebound within 4–8 weeks, even when substantial initial reductions in viral load were achieved [20, 22–25, 27, 28]. The superiority of combination regimens likely reflects their broader epitope coverage and non-overlapping resistance profiles, which together reduce the likelihood that emerging variants can escape all administered antibodies [37, 39, 44]. Baseline viral sensitivity emerged as a critical determinant of treatment success; in several trials, individuals harboring latent reservoir viruses resistant to one or more region components experienced early rebound despite achieving therapeutic serum antibody concentrations [22, 23, 25]. Moreover, maintenance of viral suppression appeared to require serum antibody concentrations above a protective threshold, often around 10 μg/ml for key V3-glycan or CD4bs-targeting antibodies [39, 44]. However, if one component declines more rapidly than the others, periods of “functional monotherapy” may arise, thereby facilitating selective viral escape and highlighting the importance of bNAb pharmacodynamic properties, neutralization breadth, and overall regimen design [38, 39, 44]. In addition, early initiation of bNAbs in acutely treated PLWH may reduce the emergence of resistance mutations in individuals with susceptible virus [29, 31]. Notably, combination of bNAbs (e.g., 3BNC117-LS, 10–1074-LS, and VRC07-523LS) with long-acting small-molecule antiretrovirals such as lenacapavir and cabotegravir has shown both excellent tolerability and promising clinical efficacy. Through week 26, one regimen demonstrated antiviral activity comparable to that of daily oral ART [46], whereas another maintained durable viral suppression in 93% of participants, with only transient infusion-related reactions [47]. Together, these findings support the development of combined long-acting antiretroviral and bNAb regimens as a promising therapeutic strategy for PLWH. Collectively, these also underscore the importance of careful patient selection, pretreatment susceptibility screening, and dosing regimens capable of sustaining protective antibody levels throughout the dosing interval.

Pharmaceutical variability further influenced the durability of antiviral control. LS-engineered variants (e.g., VRC01LS, VRC07-523LS, and PGDM1400LS) extended half-life by approximately 2- to threefold relative to their parental antibodies, thereby enabling higher peak concentrations and more sustained trough levels, which may help maintain coverage above protective thresholds [11, 13–16, 19]. These properties also support extended dosing intervals of 3–6 months, with the potential to improve adherence and retention. Nevertheless, in viremic individuals, half-life reductions of 20–40% were common, likely reflecting increased target-mediated clearance resulting from ongoing antigen-antibody complex formation, which in turn reduces systemic antibody exposure; similar mechanisms have also been reported for some anticancer antibodies [20, 22, 48]. Cross-resistance was most evident between antibodies targeting similar epitopes, such as PGT121 and 10–1074, underscoring the importance of epitope diversity in regimen design [22, 23]. Together, these observations suggest that optimizing the durability of viral control will require a dual strategy: enhancing antibody persistence through molecular engineering while tailoring combination composition regimens to baseline resistance profiles and pharmacokinetic decay patterns.

Resistance patterns may differ across individual bNAbs, even among antibodies targeting overlapping epitopes. For example, although both PGT121 and 10–1074 target the V3 glycan region, they have shown partially distinct escape profiles in rebound viruses [22], possibly reflecting differences in their binding footprints and resistance pathways. Across studies, the V3-glycan-directed antibody 10–1074 has been associated with a higher frequency of fully resistant rebound variants than the CD4-binding-site antibody 3BNC117 [23, 49], although such comparisons should be interpreted cautiously given differences in study design, viral background, and selection pressure. One possible explanation is that escape from CD4-binding-site antibodies may impose a greater fitness cost on the virus, whereas glycan-dependent epitopes targeted by antibodies such as PGDM1400 and PGT121 may be altered through glycan loss or modification with fewer structural constraints. In some studies, rapid viral rebound was observed when PGDM1400 or PGT121-resistant variants were already present at baseline, which may partly reflect the relatively high prevalence of escape-associated mutations affecting these epitopes in circulating viruses [36, 43, 50]. Overall, these findings suggest that resistance barriers differ across individual bNAbs and epitope classes, and that these differences should be taken into account when selecting antibodies for combination regimens.

Overall, bNAbs were generally well tolerated, with adverse events consisting predominantly of mild-to-moderate bNAb-related reactions, transient laboratory abnormalities, or self-limited constitutional symptoms [11–44]. Serious adverse events were rare and were not clearly attributable to the study drugs [11–44]. Immunogenicity was low across trials, with ADAs detected only in isolated cases and without measurable effects on antiviral efficacy [11–20, 24–26, 28, 29]. Likewise, GM allotype had no significant effect on the pharmacokinetic parameters for the antibodies evaluated [11, 13, 14], suggesting that host genetic factors may play only a limited role in clinical pharmacokinetic variability.

## Limitations and future directions

Despite these encouraging findings, several important challenges remain. First, the relatively small sample sizes in many studies limit statistical power and reduce the generalizability of the findings, particularly for subgroup analyses according to baseline viral sensitivity or pharmacokinetic profiles. Second, in most trials, participants initiated or resumed ART during follow-up, thereby restricting long-term evaluation of bNAb effects on viral load dynamics, latent reservoir decay, and immune responses. Third, incomplete prescreening for pre-existing resistance and the absence of systematic selection on the basis of baseline viral susceptibility in some studies may have compromised antiviral durability and facilitated early viral rebound. One currently available CLIA-certified assay for bNAb susceptibility is the Monogram HIV-1 neutralization assay. However, this assay requires approximately 4 weeks to generate results, which could delay ART initiation in viremic individuals. Moreover, in individuals receiving suppressive ART, assay results have not predicted virologic outcomes after bNAb administration, thereby limiting their clinical utility. Fourth, in several trials, amplification of pre- and post-infusion viral clones was unsuccessful in all participants, precluding full characterization of resistance emergence. Fifth, not all participants underwent plasma antiretroviral drug level testing to confirm the absence of concurrent ART use, introducing potential confounding. Sixth, most analyses were restricted to peripheral blood; the lack of pharmacokinetic and virologic data from lymphoid tissues, the central nervous system, and other tissue reservoirs limited comprehensive interpretation of reservoir dynamics and of the true exposure of the virus to bNAbs. Seventh, it remains unclear whether the observed increases in HIV-1-specific cellular immunity following bNAb administration contributed meaningfully to virologic control. Eighth, the effects of bNAb therapy on viral reservoir dynamics require further investigation in both early/acute and chronic infection. In addition, although the bNAbs evaluated in these trials bind and neutralize free HIV-1 virions effectively, their ability to eliminate infected cells may be limited. Efficient clearance of infected cells depends on Fc-mediated effector functions after antibodies bind to viral Env expressed on the cell surface. However, the current generation of clinical-stage bNAbs may not consistently elicit strong Fc-dependent effector activity in vivo, which could reduce ADCC- and complement-dependent cytotoxicity-mediated clearance. This limitation may partly explain why, despite high plasma concentrations, the observed reductions in viral load and reservoir size have often been modest. Collectively, these limitations underscore the need for future trials incorporating larger sample sizes, rigorous baseline resistance testing, longer off-ART follow-up, comprehensive tissue sampling, and integrated virologic, immunologic, and reservoir-based analyses to more fully define the therapeutic potential of bNAbs.

Advancing bNAb-based strategies toward durable HIV-1 remission will require addressing several key gaps. (i), larger, well-powered studies with extended follow-up should incorporate rigorous baseline resistance screening and standardized reservoir quantification across blood and tissue compartments. (ii), thoughtfully designed combination regimens should aim to improve delivery methods, reduce immunogenicity, and optimize dosing schedules to maximize synergy while minimizing redundancy. Such regimens may incorporate bispecific or trispecific molecules, capsid inhibitors (e.g., lenacapavir), long‑acting injectables (e.g., cabotegravir/rilpivirine), maturation inhibitors (e.g., GSK3640254), CAR‑T cells, and gene‑therapy approaches. Importantly, they must prioritize complementary resistance profiles and optimized pharmacokinetics, including LS or other half‑life‑extending modifications [51]. (iii), intermittent infusion schedules every 3–6 months, either alone or in combination with long-acting ART, merit systematic evaluation as a feasible alternative to daily oral therapy. (ⅳ), to improve the feasibility of bNAb-based strategies in routine care, a rapid point-of-care assay for bNAb sensitivity will be needed. Ideally, such an assay should provide clinically actionable results within a short timeframe and should be validated for predicting outcomes in both viremic and ART-suppressed individuals. High-throughput phenotypic approaches and emerging microfluidic neutralization platforms may represent promising directions for future development. (v), integration of immune-monitoring platforms to clarify the role of CD8^+^ T cell responses, together with structured ART re-initiation protocols after bNAb therapy to mitigate resistant rebound, will be essential. (vi), future studies incorporating tissue samples from lymphoid sites, the central nervous system, and other reservoirs for bNAb-specific pharmacokinetic and virologic assessments will be critical for elucidating the interplay among drug penetration, viral persistence, and cure strategies. Finally, future approaches combining structure‑based immunogen design, such as targeting the conserved fusion peptide and engineering Env immunogens, with next‑generation engineered antibodies, including Fc‑engineered bNAbs, multispecific or bispecific formats, and long‑acting delivery platforms, may improve infected‑cell clearance while preserving favorable pharmacokinetics [52]. Collectively, these advances could help transform bNAbs from a powerful experimental tool into a scalable component of HIV-1 remission and prevention strategies.

## Conclusions

This systematic review provides the most comprehensive clinical synthesis to date of intravenously administered bNAbs for HIV‑1 therapy. Rationally designed bNAb combinations incorporating half‑life‑extending modifications may achieve deeper and more durable viral suppression, delay viral rebound, and reduce the emergence of resistance. Baseline viral sensitivity, dosing strategies that maintain protective antibody concentrations, and careful patient selection appear to be key determinants of treatment efficacy. However, because most currently available data are derived from small early‑phase trials, these findings should be interpreted as provisional. Realizing the clinical potential of bNAbs will require larger, rigorously designed studies incorporating continued optimization of antibody design, pharmacokinetic evaluation, resistance profiling, immune monitoring, and assessment of viral reservoir dynamics. Only through such comprehensive investigations can the role of bNAbs in long‑term HIV‑1 control and remission strategies be more clearly defined.

## Supplementary Information

Additional file 1

Additional file 2

Additional file 3

Additional file 4

Additional file 5

Additional file 6

Additional file 7

Additional file 8

Additional file 9

Additional file 10

## Data Availability

No datasets were generated or analysed during the current study.

## Abbreviations

ART: Antiretroviral therapy
HIV-1: Human immunodeficiency virus type 1
VL: Viral load
bNAbs: Broadly neutralizing antibodies
AIDS: Acquired immunodeficiency syndrome
PLWH: People living with HIV-1
Env: Envelope
ADCC: Antibody-dependent cellular cytotoxicity
IgG GM: Gamma marker
ADA: Anti-drug antibody
ATI: Analytic treatment interruption
IC 50, IC 80, IC90: Inhibitory concentration 50, 80, or 90%
RCTs: Randomized controlled trials
t_1/2_: Half-life
Cmax: Peak plasma concentrations
AEs: Adverse events
