# Supplementary material for: Broadly neutralizing antibodies for HIV therapy in clinical trials: a systematic review
Source: Infect Dis Poverty. 2026 Jul 2;15:75. doi: 10.1186/s40249-026-01471-4 (PMC13326377; doi:10.1186/s40249-026-01471-4)
Supplement: Supplementary file 1 — Additional file 1 [file 40249_2026_1471_MOESM1_ESM.doc]

**Table S8A**. Frequency of local and systemic reactogenicity symptoms in PLWH receiving individual bNAbs

| **First Author** | **bNAbs** | ***n*** | **Local symptoms** | |  | **Systemic symptoms** | | | | | | |
| --- | --- | --- | --- | --- | --- | --- | --- | --- | --- | --- | --- | --- |
|  |  |  | **Pain/**  **tenderness** | **Bruising** |  | **Malaise** | **Myalgia** | **Headache** | **Chills** | **Nausea** | **Temperature** | **Joint pain** |
| Caskey M^20^ | 3BNC117 | 12@^ | 1 | 0 |  | 4 | 0 | 1 | 1 | 0 | 0 | 0 |
|  |  | 17&^ | 2 | 0 |  | 3 | 2 | 5 | 1 | 1 | 2 | 0 |
| Stephenson KE^22^ | PGT121 | 4@$ | 2 | 0 |  | 1 | 2 | 2 | 0 | 0 | 0 | 0 |
|  |  | 12@^ | 9 | 0 |  | 2 | 2 | 3 | 2 | 2 | 0 | 1 |
|  |  | 3&$ | 2 | 0 |  | 0 | 0 | 0 | 0 | 0 | 0 | 0 |
|  |  | 25&^ | 7 | 0 |  | 2 | 0 | 5 | 0 | 0 | 1 | 0 |
| Caskey M^23^ | 10-1074 | 14@^ | 0 | 0 |  | 2 | 0 | 5 | 0 | 0 | 1 | 0 |
|  |  | 19&^ | 0 | 0 |  | 0 | 0 | 3 | 0 | 1 | 0 | 2 |
| Lynch RM ^24^ | VRC01 | 20 | 1 | 0 |  | 4 | 6 | 6 | 0 | 2 | 1 | 3 |
| Happe M^25^ | VRC01LS | 7 | 0 | 0 |  | 0 | 0 | 1 | 1 | 1 | 0 | 0 |
|  | VRC07-523LS | 9 | 3 | 2 |  | 2 | 1 | 1 | 0 | 0 | 0 | 0 |
| Riddler SA ^26^ | VRC01 | 40 | NA | NA |  | NA | NA | NA | NA | NA | NA | NA |
| Scheid JF^27^ | 3BNC117 | 13 | NA | NA |  | NA | NA | NA | NA | NA | NA | NA |
| Bar KJ^28^ | VRC01 | 24 | NA | NA |  | NA | NA | NA | NA | NA | NA | NA |
| Crowell TA^29^ | VRC01 | 18 | NA | NA |  | NA | NA | NA | NA | NA | NA | NA |
| Gunst JD^31^ | 3BNC117 | 30 | NA | NA |  | NA | NA | NA | NA | NA | NA | NA |
| Cohen YZ^33^ | 3BNC117 | 15 | NA | NA |  | NA | NA | NA | NA | NA | NA | NA |
| Leone PA^34^ | N6LS | 46&^ | 2 | 2 |  | 0 | 0 | 1 | 0 | 0 | 0 | 0 |

Note: *PLWH*, people living with HIV-1; *NA*, not available. *bNAb,* broadly neutralizing antibody. The study involves analyzing data from @ (Healthy subjects), & (HIV-1 positive subjects), who received either $ (placebo) or ^ bNAb.

**Table S8B. Frequency of severity of local and systemic reactogenicity symptoms in PLWH receiving individual bNAbs.**

| **First Author** | **bNAbs** | ***n*** | **Local reactogenicity severity** | | | | |  | **Systemic reactogenicity severity** | | | |
| --- | --- | --- | --- | --- | --- | --- | --- | --- | --- | --- | --- | --- |
|  |  |  | **Total** | **Mild** | **Moderate** | | **Severe** |  | **Total** | **Mild** | **Moderate** | **Severe** |
| Caskey M^20^ | 3BNC117 | 12@^ | NA | NA | | NA | NA |  | NA | NA | NA | NA |
|  |  | 17&^ | NA | NA | | NA | NA |  | NA | NA | NA | NA |
| Stephenson KE^22^ | PGT121 | 4@$ | 2 | 2 | | 0 | 0 |  | 5 | 3 | 2 | 0 |
|  |  | 12@^ | 9 | 8 | | 1 | 0 |  | 12 | 9 | 3 | 0 |
|  |  | 3&$ | 2 | 2 | | 0 | 0 |  |  |  |  | 0 |
|  |  | 25&^ | 7 | 6 | | 1 | 0 |  | 8 | 5 | 3 | 0 |
| Caskey M^23^ | 10-1074 | 14@^ | NA | NA | | NA | NA |  | NA | NA | NA | 0 |
|  |  | 19&^ | NA | NA | | NA | NA |  | NA | NA | NA | 1 |
| Happe M^25^ | VRC01LS | 7 | 0 | 0 | | 0 | 0 |  | 3 | 3 | 0 | 0 |
|  | VRC07-523LS | 9 | 5 | 5 | | 0 | 0 |  | 4 | 4 | 0 | 0 |
| Riddler SA ^26^ | VRC01 | 40 | NA | NA | | NA | NA |  | NA | NA | NA | 0 |
| Scheid JF^27^ | 3BNC117 | 13 | NA | NA | | NA | NA |  | NA | NA | NA | 3 |
| Bar KJ^28^ | VRC01 | 24 | NA | NA | | NA | NA |  | NA | NA | NA | NA |
| Crowell TA^29^ | VRC01 | 18 | NA | NA | | NA | NA |  | NA | NA | NA | NA |
| Gunst JD^31^ | 3BNC117 | 30 | NA | NA | | NA | NA |  | NA | NA | NA | NA |
| Cohen YZ^33^ | 3BNC117 | 15 | NA | NA | | NA | NA |  | NA | NA | NA | NA |
| Leone PA^34^ | N6LS | 46&^ | 5 | NA | | NA | NA |  | NA | NA | NA | NA |

Note: *PLWH*, people living with HIV-1; *NA*, not available. *bNAb*, broadly neutralizing antibody. The study involves analyzing data from @ (Healthy subjects), & (HIV-1 positive subjects), who received either $ (placebo) or ^ bNAb.
