# Supplementary material for: Broadly neutralizing antibodies for HIV therapy in clinical trials: a systematic review
Source: Infect Dis Poverty. 2026 Jul 2;15:75. doi: 10.1186/s40249-026-01471-4 (PMC13326377; doi:10.1186/s40249-026-01471-4)
Supplement: Supplementary file 2 — Additional file 2 [file 40249_2026_1471_MOESM2_ESM.doc]

**Table S9A. Frequency of local and systemic reactogenicity symptoms in PLWH receiving combination bNAbs**

| **First Author** | **bNAbs** | ***n*** | **Local symptoms** | |  | **Systemic symptoms** | | | | | | | |
| --- | --- | --- | --- | --- | --- | --- | --- | --- | --- | --- | --- | --- | --- |
|  |  |  | **Pain/**  **tenderness** | **Bruising** |  | **Malaise** | **Myalgia** | **Headache** | **Chills** | **Nausea** | **Temperature** | | **Joint pain** |
| Bar-On Y^35^ | 3BNC117+10–1074 | 15 | NA | NA |  | NA | NA | NA | NA | NA | NA | NA | |
| Julg B^36^ | PGDM1400, PGT121, VRC07-523LS | 6@$ | 1 | 0 |  | 0 | 0 | 0 | 0 | 0 | 0 | 0 | |
|  |  | 18@^ | 10 | 0 |  | 2 | 3 | 5 | 1 | 2 | 0 | 1 | |
|  |  | 5&^ | 0 | 0 |  | 0 | 0 | 0 | 0 | 0 | 0 | 0 | |
| Sneller MC ^37^ | 3BNC117+ 10–1074 | 19 | NA | NA |  | NA | 0 | NA | NA | NA | NA | NA | |
| Gunst JD^38^ | 3BNC117+ 10–1074 | 23 | NA | NA |  | NA | 1 | NA | NA | NA | NA | NA | |
| Mendoza P^39^ | 3BNC117+ 10–1074 | 15 | NA | NA |  | NA | 4 | NA | NA | NA | NA | NA | |
| Niessl J40 | 3BNC117+ 10–1074 | 9 | NA | NA |  | NA | NA | NA | NA | NA | NA | NA | |
| Shapiro RL^41^ | VRC01LS+10–1074 | 28 | NA | NA |  | NA | NA | NA | NA | NA | NA | NA | |
| Julg B^43^ | PGT121 + VRC07-523LS×1 dose | 3@^ | 0 | 0 |  | 1 | 0 | 1 | 0 | 2 | 0 | 1 | |
|  | PGT121 + VRC07-523LS + PGDM1400×1 dsoe | 3@^ | 0 | 0 |  | 2 | 0 | 1 | 0 | 0 | 0 | 0 | |
|  | PGT121 + VRC07-523LS + PGDM1400×3 doses | 4&^ | 0 | 0 |  | 2 | 0 | 1 | 0 | 0 | 0 | 0 | |
|  | PGT121 + VRC07-523LS + PGDM1400×6 doses | 8&^ | 0 | 0 |  | 1 | 1 | 2 | 0 | 1 | 0 | 1 | |
| Gaebler C^44^ | 3BNC117+10-1074 | 18 | NA | NA |  | NA | NA | NA | NA | NA | NA | NA | |

Note: *PLWH*, people living with HIV-1; *NA*, not available. *bNAb*, broadly neutralizing antibody. The study involves analyzing data from @ (Healthy subjects), & (HIV-1 positive subjects), who received either $ (placebo) or ^ bNAb.

**Table S9B. Frequency of severity of local and systemic reactogenicity symptoms in PLWH receiving combination bNAbs.**

| **First Author** | **bNAbs** | ***n*** | **Local reactogenicity severity** | | | |  | **Systemic reactogenicity severity** | | | | |
| --- | --- | --- | --- | --- | --- | --- | --- | --- | --- | --- | --- | --- |
|  |  |  | **Total** | **Mild** | **Moderate** | **Severe** |  | **Total** | **Mild** | **Moderate** | | **Severe** |
| Bar-On Y^34^ | 3BNC117+10–1074 | 15 | NA | NA | NA | NA |  | NA | NA | NA | 0 | |
| Julg B^35^ | PGDM1400, PGT121, VRC07-523LS | 6@$ | 1 | 1 | 0 | 0 |  | 0 | 0 | 0 | 0 | |
|  |  | 18@^ | 10 | 10 | 0 | 0 |  | 14 | 4 | 10 | 0 | |
|  |  | 5&^ | 0 | 0 | 0 | 0 |  | 0 | 0 | 0 | 0 | |
| Sneller MC ^36^ | 3BNC117+ 10–1074 | 19 | NA | NA | NA | NA |  | NA | NA | NA | NA | |
| Gunst JD^37^ | 3BNC117+ 10–1074 | 23 | NA | NA | NA | NA |  | NA | NA | NA | NA | |
| Mendoza P^38^ | 3BNC117+ 10–1074 | 15 | NA | NA | NA | NA |  | NA | NA | NA | NA | |
| Niessl J^39^ | 3BNC117+ 10–1074 | 9 | NA | NA | NA | NA |  | NA | NA | NA | NA | |
| Shapiro RL^40^ | VRC01LS+10–1074 | 28 | NA | NA | NA | NA |  | NA | NA | NA | NA | |
| Julg B^41^ | PGT121 + VRC07-523LS×1 dose | 3@^ | 0 | 0 | 0 | 0 |  | 5 | 3 | 2 | 0 | |
|  | PGT121 + VRC07-523LS + PGDM1400×1 dsoe | 3@^ | 0 | 0 | 0 | 0 |  | 3 | 3 | 0 | 0 | |
|  | PGT121 + VRC07-523LS + PGDM1400×3 doses | 4&^ | 0 | 0 | 0 | 0 |  | 3 | 1 | 0 | 2 | |
|  | PGT121 + VRC07-523LS + PGDM1400×6 doses | 8&^ | 0 | 0 | 0 | 0 |  | 6 | 5 | 1 | 0 | |
| Gaebler C^42^ | 3BNC117+10-1074 | 18 | NA | NA | NA | NA |  | NA | NA | NA | NA | |

Note: *NA*, not available. *bNAb*, broadly neutralizing antibody. The study involves analyzing data from @ (Healthy subjects), & (HIV-1 positive subjects), who received either $ (placebo) or ^ bNAb.
