# Supplementary figures and images for: Broadly neutralizing antibodies for HIV therapy in clinical trials: a systematic review
Source: Infect Dis Poverty. 2026 Jul 2;15:75. doi: 10.1186/s40249-026-01471-4 (PMC13326377; doi:10.1186/s40249-026-01471-4)

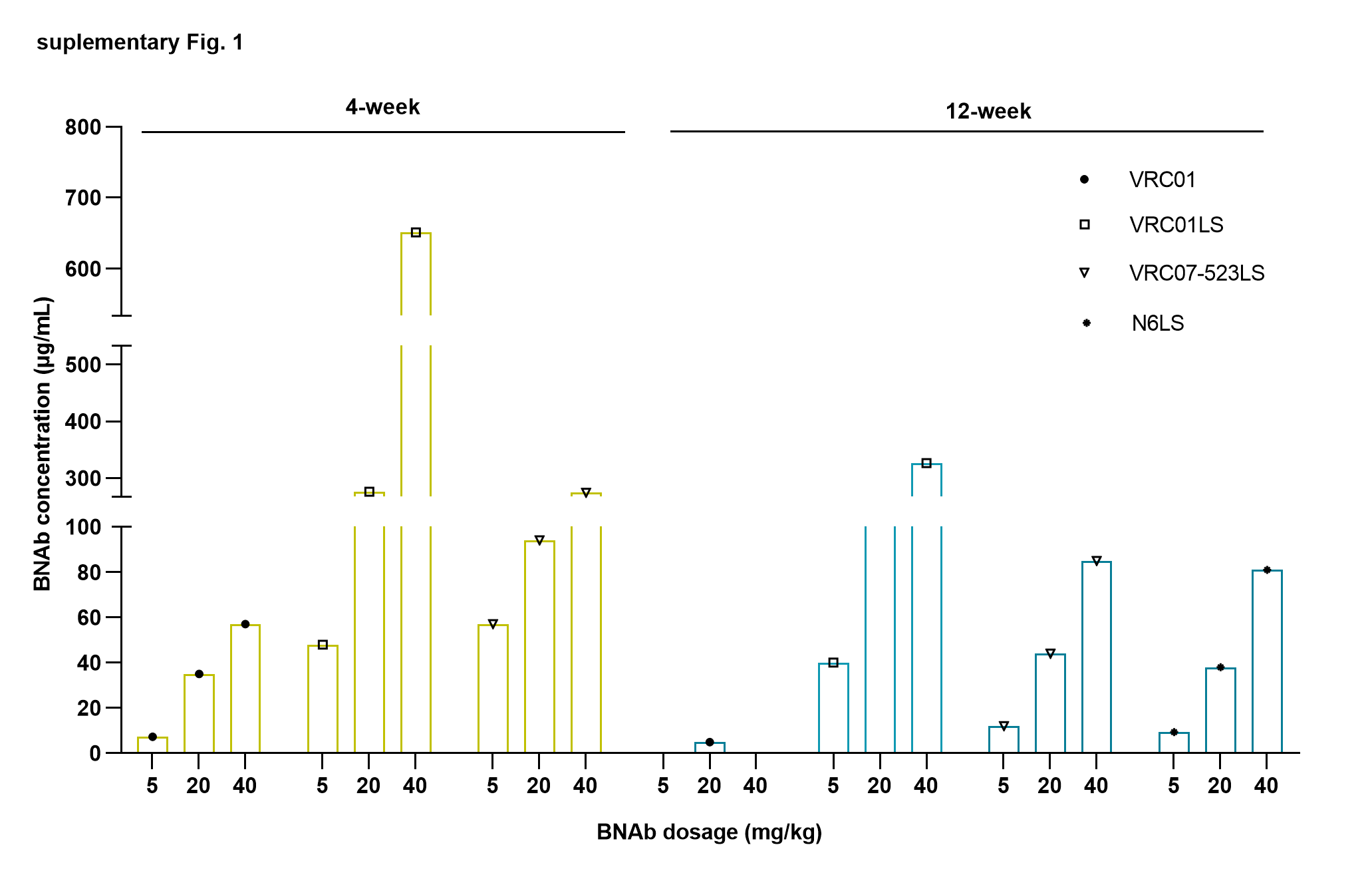

Supplement: Supplementary file 3 — Additional file 3 [file 40249_2026_1471_MOESM3_ESM.tif]
