# Supplementary material for: Broadly neutralizing antibodies for HIV therapy in clinical trials: a systematic review
Source: Infect Dis Poverty. 2026 Jul 2;15:75. doi: 10.1186/s40249-026-01471-4 (PMC13326377; doi:10.1186/s40249-026-01471-4)
Supplement: Supplementary file 4 — Additional file 4 [file 40249_2026_1471_MOESM4_ESM.doc]

**Table S1: Search strategy**

| **Database** | **Search strategy** | **Results** |
| --- | --- | --- |
| PubMed | **("HIV"[Title] OR "human immunodeficiency virus"[Title] OR "AIDS"[Title] OR "acquired immunodeficiency syndrome"[Title]) AND ("antibod*"[Title/Abstract]) NOT ("Case Reports"[Publication Type] OR "Comment"[Publication Type] OR "Editorial"[Publication Type] OR "Review"[Publication Type] OR "Meta-analysis"[Publication Type]))) AND (Clinical Trial[Publication Type])**  Up to 22 March 2026 | 1019 |
| Embase | ('human immunodeficiency virus'/exp OR HIV/exp OR HIV:ti,ab OR 'human immunodeficiency virus':ti,ab OR aids:ti,ab OR 'acquired immunodeficiency syndrome':ti,ab) AND (('monoclonal antibody'/exp OR 'monoclonal antibody':ti,ab) OR ('neutralizing antibody'/exp OR 'neutralizing antibody':ti,ab) OR ('broadly neutralizing antibody'/exp OR 'broadly neutralizing antibody':ti,ab) OR bnab*:ti,ab) AND ([humans]/lim) AND (('phase 1 clinical trial'/exp OR 'phase 2 clinical trial'/exp) OR ([clinical trial]/lim)) AND ([article]/lim)  Up to 22 March 2026 | 510 |
| Web Of Science | TS=("HIV-1" OR "human immunodeficiency virus" OR AIDS OR "acquired immunodeficiency syndrome") AND TS=("broadly neutralizing antibody" OR "neutralizing antibody" OR "monoclonal antibody" OR "bNAb") AND TS=("clinical trial" OR "phase 1" OR "phase I" OR "phase 2" OR "phase II") NOT DT=("Meeting Abstract" OR "Editorial Material" OR " Letter")  Up to 22 March 2026 | 368 |
