# Supplementary material for: Broadly neutralizing antibodies for HIV therapy in clinical trials: a systematic review
Source: Infect Dis Poverty. 2026 Jul 2;15:75. doi: 10.1186/s40249-026-01471-4 (PMC13326377; doi:10.1186/s40249-026-01471-4)
Supplement: Supplementary file 5 — Additional file 5 [file 40249_2026_1471_MOESM5_ESM.doc]

**Table S2A.** **Assessment of the quality of included studies (**based on NIH Quality Assessment Tool)

| **Author (Year)** | **Clear study question/objective** | **Defined inclusion/exclusion criteria** | **Consecutive case enrollment** | **Prospective data collection** | **Defined population** | **Clear exposure/intervention description** | **Clearly defined outcomes & valid measurement** | **Adequate follow‑up** | **Appropriate statistical analysis** | **Overall Methodological Quality** |
| --- | --- | --- | --- | --- | --- | --- | --- | --- | --- | --- |
| Ledgerwood, J E^11^ (2015) | Yes | Yes | No/NA | Yes | Yes | Yes | Yes | Yes | Yes | good |
| Mayer KH ^12^  (2017) | Yes | Yes | No | Yes | Yes | Yes | Yes | Yes | Yes | good |
| Gaudinski MR^13^ (2018) | Yes | Yes | No | Yes | No | Yes | Yes | Yes | Yes | good |
| Gaudinski MR^14^ (2019) | Yes | Yes | No/NA | Yes | Yes | Yes | Yes | Yes | Yes | good |
| Sobieszczyk ME^15^  (2023) | Yes | Yes | No/NA | Yes | Yes | Yes | Yes | Yes | Yes | good |
| Edupuganti S^16^ (2025) | Yes | Yes | No/NA | Yes | Yes | Yes | Yes | Yes | Yes | good |
| Walsh SR^17^  (2024) | Yes | Yes | No/NA | Yes | Yes | Yes | Yes | Yes | Yes | good |
| Wu RL^18^ (2025) | Yes | Yes | No/NA | Yes | Yes | Yes | Yes | Yes | Yes | good |
| Seaton KE^19^ (2025) | Yes | Yes | No/NA | Yes | Yes | Yes | Yes | Yes | Yes | good |
| Caskey M^20^ (2015) | Yes | Yes | NA | Yes | Yes | Yes | Yes | Yes | Yes | good |
| Schoofs T^21^ (2016) | Yes | Yes | NA | Yes | Yes | Yes | Yes | Yes | Yes | good |
| Stephenson KE^22^ (2021) | Yes | Yes | No/NA | Yes | Yes | Yes | Yes | Yes | Yes | good |
| Caskey M^23^ (2017) | Yes | Yes | No/NA | Yes | Yes | Yes | yes | Yes | Yes | good |
| Lynch RM^24^ (2015) | Yes | Yes | NA | Yes | Yes | Yes | yes | Yes | Yes | good |
| Happe M^25^ (2025) | Yes | Yes | NA | Yes | Yes | Yes | Yes | Yes | Yes | good |
| Riddler SA^26^ (2018) | Yes | Yes | No/NA | Yes | Yes | Yes | Yes | Yes | Yes | good |
| Scheid JF^27^ (2016) | Yes | Yes | No/NA | Yes | Yes | Yes | Yes | Yes | Yes | good |
| Bar KJ^28^ (2016) | Yes | Yes | No/NA | Yes | Yes | Yes | Yes | Yes | Yes | good |
| Crowell TA^29^ (2019) | Yes | Yes | No/NA | Yes | Yes | Yes | Yes | Yes | Yes | good |
| Gunst JD^31^ (2022) | Yes | Yes | No/NA | Yes | Yes | Yes | Yes | Yes | Yes | good |
| Cohen YZ^33^ (2018) | Yes | Yes | No/NA | Yes | Yes | Yes | Yes | Yes | Yes | good |
| Leone PA^34^ (2025) | Yes | Yes | No/NA | Yes | Yes | Yes | Yes | Yes | Yes | good |
| Bar-On Y^35^ (2018) | Yes | Yes | No/NA | Yes | Yes | Yes | Yes | Yes | Yes | good |
| Julg B^36^  (2022) | Yes | Yes | No/NA | Yes | Yes | Yes | Yes | Yes | Yes | good |
| Sneller MC^37^  (2022) | Yes | Yes | No/NA | Yes | Yes | Yes | Yes | Yes | Yes | good |
| Gunst JD^38^ (2023) | Yes | Yes | No/NA | Yes | Yes | Yes | Yes | Yes | Yes | good |
| Mendoza P^39^ (2018) | Yes | Yes | No/NA | Yes | Yes | Yes | Yes | Yes | Yes | good |
| Niessl J^40^  (2020) | Yes | Yes | No/NA | Yes | Yes | Yes | Yes | Yes | Yes | good |
| Shapiro RL^41^  (2023) | Yes | Yes | No/NA | Yes | Yes | Yes | Yes | Yes | Yes | good |
| Julg B^43^ (2024) | Yes | Yes | No/NA | Yes | Yes | Yes | Yes | Yes | Yes | good |
| Gaebler C^44^  (2022) | Yes | Yes | Yes | Yes | Yes | Yes | Yes | Yes | Yes | good |

Note: *NA*, not available.

**Table S2B. Risk of bias assessment of included studies (based on the Cochrane Risk of Bias 2 tool)**

| **Author (Year)** | **Randomisation process** | **Deviations from intended interventions** | **Missing outcome data** | **Measurement of the outcome** | **Selection of reported results** | **Overall RoB Judgment** |
| --- | --- | --- | --- | --- | --- | --- |
| Mayer KH ^12^ (2017) | low | low | low | low | low | low |
| Sobieszczyk ME^15^ (2023) | low | low | low | low | low | low |
| Edupuganti S^16^ (2025) | low | low | low | low | low | low |
| Walsh SR^17^ (2024) | low | low | low | low | low | low |
| Seaton KE^19^ (2025) | low | low | low | low | low | low |
| Stephenson KE^22^ (2021) | low | low | low | low | low | low |
| Happe M^25^ (2025) | low | low | low | low | low | low |
| Riddler SA^26^ (2018) | low | low | low | low | low | low |
| Crowell TA^29^ (2019) | low | low | low | low | low | low |
| Gunst JD^31^ (2022) | low | low | low | low | low | low |
| Leone PA^34^ (2025) | low | low | low | low | low | low |
| Sneller MC^37^ (2022) | low | low | low | low | low | low |
| Gunst JD^38^ (2023) | low | low | low | low | low | low |
| Gaebler C^44^ (2022) | low | low | low | low | low | low |

**Table S2C. Risk of bias assessment of included studies (based on the Risk of Bias in Non‑randomized Studies of Interventions tool)**

| **Author (Year)** | **Pre‑intervention: Confounding** | **Selection of participants** | **Selection into intervention** | **Deviations from intended interventions** | **Missing data** | **Measurement of outcomes** | **Selection of reported result** | **Overall ROBINS-I judgment** |
| --- | --- | --- | --- | --- | --- | --- | --- | --- |
| Ledgerwood, J E^11^ (2015) | low | low | low | low | low | low | low | low |
| Gaudinski MR^13^ (2018) | low | low | low | low | low | low | low | low |
| Gaudinski MR^14^ (2019) | low | low | low | low | low | low | low | low |
| Wu RL^18^ (2025) | low | low | low | low | low | low | low | low |
| Seaton KE^20^ (2025) | moderate | low | low | low | low | low-moderate | low | moderate |
| Schoofs T^21^ (2016) | moderate | low | low | low | low | moderate | low | moderate |
| Caskey M^23^ (2017) | moderate | low | low | low | low | low | low | moderate |
| Lynch RM^24^ (2015) | serious | moderate | low | low | low | low | low | serious |
| Scheid JF^27^ (2016) | serious | moderate | low | low | low | low | low | serious |
| Bar KJ^28^ (2016) | serious | moderate | low | low | low | low | low | serious |
| Cohen YZ^33^ (2018) | moderate | low | low | low | low | low | low | moderate |
| Bar-On Y^35^ (2018) | serious | moderate | low | low | low | low | low | serious |
| Julg B^36^  (2022) | serious | moderate | low | low | moderate | low | low | serious |
| Mendoza P^39^ (2018) | moderate | low | low | moderate | low | low | low | moderate |
| Niessl J^40^  (2020) | moderate | moderate | low | low | low | low | low | moderate |
| Shapiro RL^41^  (2023) | serious | moderate | low | low | low | low | low | serious |
| Julg B^43^ (2024) | serious | low | low | low | low | low | low | serious |
