# Supplementary material for: Broadly neutralizing antibodies for HIV therapy in clinical trials: a systematic review
Source: Infect Dis Poverty. 2026 Jul 2;15:75. doi: 10.1186/s40249-026-01471-4 (PMC13326377; doi:10.1186/s40249-026-01471-4)
Supplement: Supplementary file 6 — Additional file 6 [file 40249_2026_1471_MOESM6_ESM.doc]

**Table S3.** **Certainty assessment (**based on the Grading of Recommendations Assessment)

| **Author (Year)** | **Safety** | **Pharmacokinetics** | **Neutralisation (in‑vitro)** | **Viraemia suppression** | **Reservoir dynamics** | **Immune response** |
| --- | --- | --- | --- | --- | --- | --- |
| Ledgerwood, J E^11^ (2015) | moderate | moderate | low | NA | NA | NA |
| Mayer KH ^12^ (2017) | moderate | moderate | low | NA | NA | NA |
| Gaudinski MR^13^ (2018) | moderate | moderate | low | NA | NA | NA |
| Gaudinski MR^14^ (2019) | moderate | moderate | low | NA | NA | NA |
| Sobieszczyk ME^15^ (2023) | moderate | moderate | low | NA | NA | NA |
| Edupuganti S^16^ (2025) | moderate | moderate | low | NA | NA | NA |
| Walsh SR^17^ (2024) | moderate | moderate | low | NA | NA | NA |
| Wu RL^18^ (2025) | moderate | moderate | low | NA | NA | NA |
| Seaton KE^19^ (2025) | moderate | moderate | low | NA | NA | NA |
| Caskey M^20^ (2015) | very low | loaw | NA | low | NA | NA |
| Schoofs T^21^ (2016) | very low | NA | NA | low | NA | low |
| Stephenson KE^22^ (2021) | moderate | high | NA | moderate | NA | low |
| Caskey M^23^ (2017) | very low | low | NA | moderate | NA | low |
| Lynch RM^24^ (2015) | very low | high | NA | moderate | NA | NA |
| Happe M^25^ (2025) | very low | low | very low | moderate | NA | low |
| Riddler SA^26^ (2018) | moderate | high | NA | low | low | NA |
| Scheid JF^27^ (2016) | moderate | high | NA | moderate | NA | low |
| Bar KJ^28^ (2016) | moderate | high | NA | high | NA | low |
| Crowell TA^29^ (2019) | moderate | high | NA | high | NA | low |
| Gunst JD^31^ (2022) | moderate | NA | NA | high | high | low |
| Rosás-Umbert M^32^ (2022) | NA | NA | NA | NA | NA | high |
| Leone PA^34^ (2025) | moderate | high | NA | low | NA | low |
| Bar-On Y^35^ (2018) | moderate | high | NA | low | NA | low |
| Julg B^36^ (2022) | moderate | high | NA | low | NA | low |
| Sneller MC^37^ (2022) | moderate | NA | NA | very high | NA | low |
| Gunst JD^38^ (2023) | moderate | high | NA | moderate | high | low |
| Mendoza P^39^ (2018) | moderate | high | NA | very high | NA | low |
| Niessl J^40^ (2020) | moderate | NA | NA | low | NA | high |
| Shapiro RL^41^ (2023) | moderate | high | NA | very high | NA | low |
| Niesar A^42^ (2026) | NA | NA | NA | NA | NA | moderate |
| Julg B^43^ (2024) | very low | high | NA | moderate | NA | low |
| Gaebler C^44^ (2022) | moderate | high | NA | high | NA | low |

*NA*, not available.
