# Supplementary material for: Broadly neutralizing antibodies for HIV therapy in clinical trials: a systematic review
Source: Infect Dis Poverty. 2026 Jul 2;15:75. doi: 10.1186/s40249-026-01471-4 (PMC13326377; doi:10.1186/s40249-026-01471-4)
Supplement: Supplementary file 7 — Additional file 7 [file 40249_2026_1471_MOESM7_ESM.doc]

**Table S4. Characteristics of PLWH treated with individual bNAbs**

| **First Author** | **ADA** | **Follow-up**  **(weeks)** | **Uninfected** | | | |  | **HIV-1-infected (day0)** | | | | | | | | |
| --- | --- | --- | --- | --- | --- | --- | --- | --- | --- | --- | --- | --- | --- | --- | --- | --- |
|  |  |  | ***n*** | **Male**  **(%)** | **Age**  **(Years)** | **Hispanic**  **(%)** |  | **On ART (*n*)** | **Off ART viremic (*n*)** | **Naïve**  **Viremic**  **(*n*)** | **Male**  **(%)** | **Age**  **(Years)** | **Hispanic**  **(%)** | **CD4^+^count**  **(median, range)** | **HIV-1 RNA**  **(copies/ml)** | **Time on ART (years)** |
| Caskey M^20^ | No | 8 | 12 | 83 | 43* (22, 58) | 8 |  | 2 | 15 |  | 76 | 37* (20, 54) | 18 | 655* (245, 1,129) | 9,420* (470, 64053) | NA |
| Stephenson KE^22^ | 2/48 | beyond  24 | 16 | NA | (19, 48)# | NA |  | 15 | High: 9  Low: 4 |  | 89 | (20, 62) | 25 | (494, 920)# | High: 21,040 (9,660, 28,990)  Low: 270 (185, 550) | NA |
| Caskey M^23^ | NA | 24 | 14 | 71 | 43* (25, 60) | 7 |  | 3 | 16 |  | 84 | 39* (24, 53) | 11 | 593* (289, 880) | 12,851* (840, 77,610) | NA |
| Lynch RM ^24^ | No | 24 | 20 |  |  |  |  | 12 | 2 | 6 | NA | 36 (21, 64) | NA | 614 (228, 1190) | 1: 23; 8: 5080 (237, 27,894) | NA |
| Happe M^25^ | No | 48 |  |  |  |  |  |  | 1 | 15 | 71;100 | 39 (25, 57); 26 (20, 56) | 0;22 | 523 (395, 817);  533 (382, 876) | 3177 (258, 174134);  20738 (12149, 70435) | NA;NA |
| Riddler SA ^26^ | No | 18 |  |  |  |  |  | 40 |  |  | 93 | 52 (41–58) | 6 | 696 (559–889) | <40 | NA |
| Scheid JF^27^ | NA | 36 |  |  |  |  |  | Group A: 6; Group B: 7 |  |  | 100;14 | 42* (26, 62); 35* (27, 57) | NA; NA | 758* (689, 958);  739* (358, 1031) | <50;  <50 | 11* (4, 18);  4* (1, 112) |
| Bar KJ^28^ | No | 15-20 |  |  |  |  |  | A5340: 14; NIH: 10 |  |  | 100;80 | 38 (27, 52);  51 (33, 59) | 14; 10 | 896 (IQR:579–1053);  724 (IQR: 630–926) | 13: <50,  1: >50;  <50 | 4.7 (IQR: 3.8–6.0);  10.0 (IQR: 7.7–13.3) |
| Crowell TA^29^ | No | 32-48 |  |  |  |  |  | Placebo: 5; VRC01: 13 |  |  | 100;100 | 25 (23-48);  32 (21-50) | NA; NA | 562 (431-735);  769 (402-1032) | <50;  <50 | 2.7 (2.3-3.8);  3.1 (2.3-6.6) |
| Gunst JD^31^ | NA | 42 |  |  |  |  |  |  |  | ART: 15; +bNAb: 15 | 73;100 | 33 (25-58);  41 (25-69) | NA; NA | 560 (252-1,497);  506 (230, 984) | 39207 (820, 3240000);  56.800 (740-24,000,000) | 0;0 |
| Cohen YZ^33^ | NA | 60 |  |  |  |  |  | 15 |  |  | 93 | 43 (26–58) | 20 | <50 | 688 (391–1,418) | 11 (1, 21) |
| Leone PA^34^ | 39% | 48 |  |  |  |  |  |  |  | 46 | 93 | 29.5*(18-61)# | 83 | (179-850)# | (126-630,960)# | 0 |

Note: *PLWH*, people living with HIV-1; *bNAb*, broadly neutralising antibody; *ART*, antiretroviral therapy; *NA*, not available. The “mean” is denoted by *, and the range is denoted by #.aa
