# Supplementary material for: Broadly neutralizing antibodies for HIV therapy in clinical trials: a systematic review
Source: Infect Dis Poverty. 2026 Jul 2;15:75. doi: 10.1186/s40249-026-01471-4 (PMC13326377; doi:10.1186/s40249-026-01471-4)
Supplement: Supplementary file 8 — Additional file 8 [file 40249_2026_1471_MOESM8_ESM.doc]

**Table S5. Characteristics of PLWH treated with combination bNAbs**

| **First Author** | **ADA** | **Follow-up**  **(weeks)** | **Uninfected HIV-1-infected (day0)** | | | |  | **HIV-1-infected (day0)** | | | | | | | | |
| --- | --- | --- | --- | --- | --- | --- | --- | --- | --- | --- | --- | --- | --- | --- | --- | --- |
|  |  |  | ***n*** | **Male**  **(%)** | **Age**  **(Year)** | **Hispanic (%)** |  | **On ART (*n*)** | **Off ART viremic *(n*)** | **Naïve**  **Viremic**  **(*n*)** | **Male**  **(%)** | **Age**  **(Years)** | **Hispanic**  **(%)** | **CD4^+^**  **count** | **HIV-1 RNA**  **(copies/ml)** | **Time on ART (years)** |
| Bar-On Y^35^ | NA | 24 |  |  |  |  |  | 8 | 2 | 5 | 73 | 45(26, 64)* | 13 | 662 (358, 1033) | < 20 | NA |
|  |  |  |  |  |  |  |  |  |  |  |  |  |  | 570* (320, 780) | 11494* (730, 97800) | NA |
| Julg B^36^ |  | 25 | 24 | 54 | (20, 49) | 8.3 |  |  | 5 |  | 100 | 38 (24, 59)* | 40 | 505 (392–575)* | 16,066 (2,770–163,130)* |  |
| Sneller MC ^37^ | NA | > 48 |  |  |  |  |  | placebo: 7 |  |  | 100 | 40 (27, 57) | 14 | 799 (543–1177) | <20 | NA |
|  |  |  |  |  |  |  |  | bNAb: 7 |  |  | 100 | 34 (29, 56) | 14 | 612 (426–832) | <20 | NA |
|  |  |  |  |  |  |  |  |  |  | bNAb: 5 | 100 | 44 (35, 52) | 0 | 640 (527–1011) | 493 (147, 2984) | NA |
| Gunst JD^38^ | NA | 25 |  |  |  |  |  | placebo:11 |  |  | 82 | 54 (45, 60) | NA | 743 (708–820) | <50 | 8 (4–23) |
|  |  |  |  |  |  |  |  | bNAb: 12 |  |  | 75 | 51 (41, 54) | NA | 1,027 (808–1,240) | <50 | 8 (2, 23) |
| Mendoza P^39^ | NA | 30 |  |  |  |  |  | 15 |  |  | 93 | 40 (22, 55) | 27 | 730 (515, 1360) | <20 | 5 (2,21) |
| Niessl J^40^ | NA | 30 |  |  |  |  |  | bNAb: 9 |  |  | 11 | 36 (29, 51) | 44.4 | 728 (350, 1360) | <20 | 5 (3, 21) |
|  |  |  |  |  |  |  |  | ART:13 |  |  | 85 | 48 (25., 59) | 8 | 715 (461, 1370) | <20 | 11 (3, 21) |
| Shapiro RL^41^ | No | 24 |  |  |  |  |  | 28 |  |  | 32 | 3.6 (2.4, 5.6) | NA | 1198 (IQR 843–1684) | 2/28:>40  26/28: <40 | at least 96 weeks |
| Julg B^43^ | No | 44 | group 1A:3^ | 33 | 25* (22, 29) | 33 |  | group 2: 4 |  |  | 75 | 40* (28, 59) | 50 | ≥400 | <50 | NA |
|  |  |  | group 1B: 3^ | 33 | 32* (23, 44) | 33 |  | group3: 8 |  |  | 75 | 42* (23, 59) | 50 | ≥400 | <50 | NA |
| Gaebler C^44^ | No | 48 |  |  |  |  |  | ART: 10 |  |  | 60 | 45 (30, 60) | 10 | 776* (480, 1221) | <50 | 15 (9, 24) |
|  |  |  |  |  |  |  |  | group1: 18 |  |  | 89 | 51 (33, 59) | 23 | 778* (545, 1192) | <50 | 10 (1, 29) |
|  |  |  |  |  |  |  |  | group2: 8 |  |  | 88 | 49 (31, 60) | 11 | 638* (483, 806) | <50 | 10 (4, 21) |

Note: *PLWH*, people living with HIV-1; *NA*, not available. The mean is denoted by *, and the range is denoted by #.
