# Supplementary material for: Broadly neutralizing antibodies for HIV therapy in clinical trials: a systematic review
Source: Infect Dis Poverty. 2026 Jul 2;15:75. doi: 10.1186/s40249-026-01471-4 (PMC13326377; doi:10.1186/s40249-026-01471-4)
Supplement: Supplementary file 9 — Additional file 9 [file 40249_2026_1471_MOESM9_ESM.doc]

**Table S6. Pharmacokinetic characteristics of bNAbs in HIV-negetive individuals and PLWH**

| **First Author** | **bNAbs** | **t_1/2_ (days)** | **C_max_ (μg/ml)** | **T_max_ (μg/ml)** | **Week-C (μg/ml)** | **C (μg/ml)** |
| --- | --- | --- | --- | --- | --- | --- |
| Ledgerwood, J E^11^ | VRC01 | Overall: 15;  14 (5 and 40 mg/kg);  17 (20 mg/kg). | After 1st dose:  210 at 5 mg/kg;  1100 at 20 mg/kg;  1500 at 40 mg/kg. | After 1st dose:  1.6 at 5 mg/kg;  2.0 at 20 mg/kg;  1.9 at 40 mg/kg. | 4-week:  10 at 5 mg/kg;  56 at 20 mg/kg;  89 at 40 mg/kg. | NA |
| Mayer KH ^12^ | VRC01 | Overall 15;  11.4 at 10 mg/kg;  13.7 at 30 mg/kg. | At 1 hour after the last dose:   420 at 10 mg/kg;  796 at 20 mg/kg;  1,177 at 30 mg/kg;  1549 at 40 mg/kg. | 56 days after the firs infusion：  20 at 40 mg/kg;  12 at 30 mg/kg;  4 at 10 mg/kg;  28 days after the first infusion:  69 at 20 mg/mg. | 1. week:   6 at 10mg/kg;  46 at 20 mg/days;  17 at 30 mg/kg;  27 at 40 mg/days. | NA |
| Gaudinski MR^13^ | VRC01LS | Overall 71；  83 at 5 mg/kg；  76 at 20 mg/kg;  55 at 40 mg/kg. | After one infusion:  246 at 5 mg/kg;  1221 at 20 mg/kg;  2234 at 40 mg/kg. | 1. 0.07 at 5mg/kg;   0.2 at 20 mg/kg;  0.05 at 40 mg/kg;   1. After infusion of 5 mg/kg, concentrations above 10 μg/ml were maintained for more than 20 weeks. | 4-week: 48 at 5 mg/kg; 276 at 20 mg/kg;  651 at 40 mg/kg;   1. week: 40 at 5 mg/kg;   180 at 20 mg/kg;  326 at 40 mg/kg. | NA |
| Gaudinski MR^14^ | VRC07–523LS | Overall: 38 | After the first infusion：  47 at 1mg/kg;  240 at 5 mg/kg;  869 at 20 mg/kg;  1630 at 40 mg/kg. | After single dose:  0.7 at 1 mg/kg;  0.04 at 5 mg/kg;  0.3 at 20 mg/kg;  0.04 at 40 mg/kg. | 4-week:  14 at 1mg/kg;  57 at 5 mg/kg;  148 at 20 mg/kg;  274 at 40 mg/kg;   1. week：   3.8 at 1 mg/kg;  12 at 5 mg/kg;  44 at 20 mg/kg;  85 at 40 mg/kg. | NA |
| Sobieszczyk ME^15^ | PTG121  VRC07–523LS  PGDM1400  10-1074 | Overall:  32.2 for PGT121;  25.4 for PGDM1400;  27.5 for 10–1074;  52.9 for VRC07–523LS. | NA | NA | After the first infusion, at 20 mg/kg,   1. week C:   51 for PGT121;  71 for PGDM1400;  238 for 10–1074;  94 for VRC07–523LS;   1. week:   15 for PGT121;  12 for PGDM1400;  NA for 10–1074;  42 for VRC07–523LS. | NA |
| Edupuganti S^16^ | PGT121.414LS  VRC07-523LS | PGT121.414.LS: 71;  VRC07-523LS: 53. | After the first infusion at 1 h,  PGT121.414.LS at 3, 10, 20, 30 mg/kg was 63.3, 221.7, 714.2, and 554.7; VRC07-523LS: 630.4 at 20 mg/kg. | NA | NA | NA |
| Walsh SR^17^ | VRC07-523LS | Overall: 42.4. | On day 3:  29.0 at 2.5 mg/kg;  58.5 at 5 mg/kg;  257.2 at 20 mg/kg. | NA | On day 112:  3.4 at 2.5 mg/kg;  6.5 at 5 mg/kg;  27.2 at 20 mg/kg. | NA |
| Wu RL^18^ | N6LS | Overall: 48.6. | 109 at 5 mg/kg;  442 at 20 mg/kg;  737 at 40 mg/kg. |  | 12 -week:  9.3 at 5 mg/kg;  38 at 20 mg/kg;  81 at 40 mg/kg. | NA |
| Seaton KE^19^ | PGDM1400LS | Overall: 55;  47.9 at 5 mg/kg;  58.8 at 20 mg/kg;  55.2 days at 40 mg/kg. | 95.7 at 5 mg/kg;  432.6 at 20 mg/kg;  727.4 at 40 mg/kg. | NA | NA | NA |
| Caskey M^20^ | 3BNC117 | HD: overall: 17;  At 3, 10, and 30 mg/kg  18.0, 18.7, and 15.3. | HD:  At 1, 3, 10, and 30 mg/kg:  19.1, 141.1, 279.1,and 495.9. | NA | NA | NA |
|  |  | Viremic PLWH: overall: 9;  At 3, 10, and 30 mg/kg  9.1, 8.1 and 10.3. | Viremic PLWH: At 1, 3, 10, and 30 mg/kg:  24.1, 73.7, 235.4, and 669.8. |  |  |  |
| Schoofs T^21^ | 3BNC117 | NA | NA | NA | NA | NA |
| Stephenson KE^22^ | PGT121 | HD: overall: 22  At 3, 10, and 30 mg/kg:  HD: 19.7, 22.8, 23.9. | HD at 3, 10, and 30 mg/kg:  52, 254, and 759. | NA | NA | NA |
|  |  | Aviremic PLWH: overall: 16.  At 3, 10, and 30 mg/kg:  13.3, 12.48, and 16.6. | Aviremic PLWH: At 3, 10, and 30 mg/kg: 54, 241, and 929. |  |  |  |
|  |  | Viremic PLWH: overall: 14.  High vs. Low: 12.5 vs. 14. | Viremic PLWH: High vs. Low: 731 vs. 648 at 30 mg/kg |  |  |  |
| Caskey M^23^ | 10-1074 | HD: overall HD: 24.  At doses of 3, 10, and 30 mg/kg: 23.9, 26.9, and 23.0. | HD at 3, 10, and 30 mg/kg:  3 mg/kg: 91.7, 364.7 and 1417.1. | NA | NA | At viral rebound: 10 mg/kg: 23.7  30 mg/kg: 76.9 |
|  |  | Viremic PLWH: 12.8;  At doses of 3, 10, and 30 mg/kg: 18.7, 11.0, and 11.9. | Viremic PLWH: at 3, 10, and 30 mg/kg:  54.5, 199.4 and 1076.1. |  |  |  |
| Lynch RM^24^ | VRC01 | 12 days in all PLWH.  Aviremic PLWH: at 1, 5, 20, and 40 mg/kg： 13, 14, 18, and 8.6. | Aviremic PLWH: at 1, 5, 20, and 40 mg/kg after 1^st^ infusion：27, 240, 1000, and 1600. | NA | NA | Day 28: Aviremic: at 1, 5, 20, and 40 mg/kg after 1^st^ infusion：1, 7.3, 33, and 34. |
|  |  | Viremic PLWH: 9.1 at 40 mg/kg. | Viremic PLWH: at 40 mg/kg after 1^st^ infusion: 1400. |  |  | Viremic: at 40 mg/kg after 1^st^ infusion: 28. |
| Happe M^25^ | VRC01LS VRC07-523LS | Viremic PLWH: VRC01LS vs. VRC07-523LS: 47.3 vs. 56.5 days. | Viremic PLWH: VRC01LS vs. VRC07-523LS: 1,566 vs. 1,295. | NA | NA | NA |
| Riddler SA^26^ | VRC01 40 | NA | NA | NA | At 3 weeks were >50 in 39/40 cases (mean 112.2 ). | NA |
| Scheid JF^27^ | 3BNC117 30 mg/kg and AIT 2d after the first infusion:  Group A: day 0 and 21;  Group B: day 0, 14, 28 and 42. | During ATI Group A vs B: 19.6 vs 14.1 days. | NA | NA |  | At viral rebound:  6–41 (mean 19.7). |
| Bar KJ^28^ | VRC01 40 mg/kg:  A5340: -1 week before and 2 and 5 weeks after ATI; ATI 1 week after the first infusion.  NIH: -3 days before ATI, 2 and 4 weeks after ATI, then monthly up to 6 months. | NA | NA | NA | A5340 maintained plasma VRC01 >50 for 8 weeks (NIH >100 throughout). | At viral rebound:  >50, except one 25. |
| Crowell TA^29^ | VRC01 | NA | NA | Consistent VRC01 concentration: >50 during dosing | NA | Plasma concentration: VRC01 at ART restart: 277. |
| Cale EM^30^ | VRC01 | NA | NA | NA | NA | NA |
| Gunst JD^31^ | 3BNC117 | NA | NA | NA | NA | NA |
| Rosás-Umbert M^32^ | 3BNC117 | NA | NA | NA | NA | NA |
| Cohen YZ^33^ |  | Aviremic PLWH: 14.7 days. | NA | NA | NA | NA |
| Leone PA^34^ | N6LS | Viremic PLWH: 24.1, 21.2, 14.5, and 9.9 days for 40, ~10, ~4 and ~1 mg/kg. | Viremic PLWH: 1132, 243, 131 and 43 for 40, ~10, ~4 and ~1 mg/kg. | NA | NA | NA |
| Bar-On Y^35^ | 3BNC117  10-1074 | 3BNC117 vs. 10-1074 Overall:   1. Viremic PLWH: ELISA: 11.1 vs 12.2; 2. Aviremic PLWH: ELISA: 14.5 vs 19, | BNC117: Aviremic PLWH: at 10 and 30 mg/kg: 225.4 and 1166.2;  Viremic PLWH: ( x1) 817.9; ( x3) 861.3; | NA | NA | NA |
|  |  | 3BNC117: Aviremic PLWH: at 10 and 30 mg/kg: 16.4 and 12.5 days;  Viremic PLWH: at 30 mg/kg  : 10.1; ( x3): 12.3; | 10-1074: Aviremic PLWH: at 10 and 30 mg/kg: 350.2 and 1172.3；  Viremic PLWH: at 30 mg/kg  ( x1): 1249.0; ( x3): 2359.4; |  |  |  |
|  |  | 10-1074: Aviremic PLWH: at 10 mg/kg and 30 mg/kg: 19.9 and 18.1;  Viremic PLWH: at 30 mg/kg  (x1): 11.9; (x3): 12.7. | Combination: no change. |  |  |  |
| Julg B^36^ | PGDM1400  PGT121  VRC07-523LS | HD: PGDM1400: 20.77 days alone; 17.4 days with PGT121; PGT121 alone: 20.2 days; | NA | NA | NA | NA |
|  |  | Viremic PLWH: PGDM1400: 11 days; PGT121: 11.8 days; VRC07-523LS: 29.3 days. |  |  |  |  |
| Sneller MC^37^ | 3BNC117  10–1074 | NA | NA | NA | NA | NA |
| Gunst JD^38^ | 3BNC117  10–1074 | NA | NA | NA | NA | Virologic failure: placebo/bNAb group, 3BNC117: 13.2; 10-1074: 73.3. |
| Mendoza P^39^ | 3BNC117  10-1074 | Aviremic PLWH: 3BNC117 and 10-1074: 17.6 and 23.2 days by ELISA. | NA | NA | NA | 3BNC117 and 10-1074 at rebound was 1.9 and 14.8, respectively. |
| Niessl J40 | 3BNC117  10-1074 | NA | NA | NA | NA | NA |
| Shapiro RL^41^ | 10–1074  VRC01LS | NA | NA | 211.0 for 10–1074 and 259.6 for VRC01LS. | NA | NA |
| Niesar A^42^ | 10–1074  VRC01LS | NA | NA | NA | NA | NA |
| Julg B^43^ | PGT121  VRC07-523LS  PGDM1400 | 1. HD, dual-bNAbs: PGT121: 20.4 days and VRC07-523LS: 37.7 days; 2. HD, triple-bNAbs: PGT121: 21.1 days, PGDM1400: 23.2 days and VRC07-523LS: 38.7 days; | NA | NA | NA | At the time of viral rebound: PGT121 and PGDM1400 < 10 and VRC07-523LS <100. |
|  |  | Aviremic PLWH, triple-bNAbs, 3-6 infusions): PGT121: 19.9 days, PGDM1400: 23.9 days and VRC07-523LS: 44.9 days. |  |  |  |  |
| Gaebler C^44^ | 3BNC117  10-1074 | Aviremic PLWH: 3BNC117 and 10-1074: 14.9 and 20.3 days. | NA | NA | NA | Rebound after week 20: 3BNC117: 3.5, 10-1074: 28.3. |

Note: *PLWH*, people living with HIV-1; *PK*, pharmacokinetics; *t_1/2_,* half-life; *Cmax*, peak plasma concentrations; *T*, trough plasma concentrations; *bNAb*, broadly neutralizing antibody.
