# Supplementary material for: Broadly neutralizing antibodies for HIV therapy in clinical trials: a systematic review
Source: Infect Dis Poverty. 2026 Jul 2;15:75. doi: 10.1186/s40249-026-01471-4 (PMC13326377; doi:10.1186/s40249-026-01471-4)
Supplement: Supplementary file 10 — Additional file 10 [file 40249_2026_1471_MOESM10_ESM.doc]

**Table S7A. Frequency of local and systemic reactogenicity symptoms in HIV-negative individuals.**

| **First Author** | **bNAbs** | ***n*** | **Local symptoms** | |  | **Systemic symptoms** | | | | | | |
| --- | --- | --- | --- | --- | --- | --- | --- | --- | --- | --- | --- | --- |
|  |  |  | **Pain/**  **tenderness** | **Bruising** |  | **Malaise** | **Myalgia** | **Headache** | **Chills** | **Nausea** | **Temperature** | **Joint pain** |
| Ledgerwood J E^11^ | VRC01 | 18 | 2 | 0 |  | 2 | 4 | 5 | 0 | 1 | 0 | 0 |
| Mayer KH^12^ | VRC01 | 64 | NA | NA |  | NA | NA | NA | NA | NA | NA | NA |
| Gaudinski MR^13^ | VRC01LS | 19 | 2 | 1 |  | 4 | 3 | 1 | 0 | 0 | 0 | 0 |
| Gaudinski MR^14^ | VRC07–523LS | 18 | 0 | 1 |  | 3 | 3 | 2 | 2 | 1 | 2 | 1 |
| Sobieszczyk ME^15^ | PTG121  VRC07–523LS  PGDM1400  10-1074 | 27 | NA | NA |  | NA | NA | NA | NA | NA | NA | NA |
| Edupuganti S^16^ | PGT121.414.LS | 10 | 0 | 0 |  | 3 | 0 | 3 | 0 | 1 | 0 | 0 |
|  | PGT121.414.LS+VRC07-523LS | 10 | 6 | 0 |  | 6 | 2 | 7 | 2 | 3 | 0 | 2 |
| Walsh SR^17^ | VRC07-523LS | 59 | NA | NA |  | NA | NA | NA | NA | NA | NA | NA |
| Wu RL^18^ | N6LS | 14 | NA | NA |  | NA | NA | NA | NA | NA | NA | NA |
| Seaton KE ^19^ | PGDM1400LS | 9 | 1 | 0 |  | 3 | 0 | 3 | 0 | 0 | 0 | 0 |

Note: *NA*, not available.

**Table S7B. Frequency of severity of local and systemic reactogenicity symptoms in HIV-negative individuals.**

| **First Author** | **bNAbs** | **n** | **Local reactogenicity severity** | | | | |  | **Systemic reactogenicity severity** | | | |
| --- | --- | --- | --- | --- | --- | --- | --- | --- | --- | --- | --- | --- |
|  |  |  | **Total** | **Mild** | **Moderate** | | **Severe** |  | **Total** | **Mild** | **Moderate** | **Severe** |
| Ledgerwood, J E^11^ | VRC01 | 18 | 2 | 2 | 0 | 0 | |  | 8 | 8 | 0 | 0 |
| Mayer KH^12^ | VRC01 | 84 | NA | NA | NA | NA | |  | NA | NA | NA | 3 |
| Gaudinski MR^13^ | VRC01LS | 19 | 3 | 3 | 0 | 0 | |  | 8 | 8 | 0 | 0 |
| Gaudinski MR^14^ | VRC07–523LS | 17 | 1 | 1 | 0 | 0 | |  | 14 | 9 | 5 | 0 |
| Sobieszczyk ME^15^ | PTG121, VRC07–523LS, PGDM1400, 10-1074 | 27 | NA | NA | NA | 0 | |  | NA | NA | NA | 1 |
| Edupuganti S^16^ | PGT121.414.LS | 10 | 0 | 0 | 0 | 0 | |  | 7 | 7 | 0 | 0 |
|  | PGT121.414.LS+VRC07-523LS | 10 | 6 | 6 | 0 | 0 | |  | 22 | 18 | 4 | 0 |
| Walsh SR^17^ | VRC07-523LS | 59 | NA | NA | NA | NA | |  | NA | NA | NA | 3 |
| Wu RL^18^ | N6LS | 14 | NA | NA | NA | NA | |  | NA | NA | NA | 3 |
| Seaton KE ^19^ | PGDM1400LS | 9 | 1 | 1 | 0 | 0 | |  | 7 | 6 | 1 | 0 |

Note: NA, not available.
